# Early mechanical changes of limb muscle in septic shock assessed by dual-plane shear-wave elastography

**DOI:** 10.1186/s40635-026-00968-4

**Published:** 2026-09-02

**Authors:** Ivo Neto Silva, Rui Azevedo, Joana Duarte, Stanislas Abrard, José Alberto Duarte, Karim Bendjelid

**Affiliations:** 1 Intensive Care Division, Department of Acute Medicine, Geneva University Hospitals, Rue Gabrielle-Perret-Gentil 4, Geneva, CH-1205 Switzerland; 2 Geneva Hemodynamic Research Group, Faculty of Medicine, University of Geneva, Geneva, Switzerland; 3 Care Directorate, Geneva University Hospitals, Geneva, Switzerland; 4 Research Centre of Physical Activity, Health and Leisure (CIAFEL), Faculty of Sport, University of Porto (FADE-UP), Porto, Portugal; 5 Associate Laboratory i4HB - Institute for Health and Bioeconomy, University Institute of Health Sciences - CESPU, Gandra, Portugal; 6 UCIBIO - Applied Molecular Biosciences Unit, Forensic Sciences Research Laboratory, University Institute of Health Sciences (1H-TOXRUN, IUCS- CESPU), Gandra, Portugal; 7 Anaesthesiology Division, Department of Acute Medicine, Geneva University Hospitals, Geneva, Switzerland; 8 Department of Anaesthesiology and Critical Care Medicine, Hospices Civils de Lyon, Edouard Herriot Hospital, Lyon, France; 9 Research on Health care Performance (RESHAPE), INSERM U1290, University Claude Bernard Lyon 1, Lyon, France; 10 UCIBIO - Applied Molecular Biosciences Unit, Translational Toxicology Research Laboratory, University Institute of Health Sciences (1H-TOXRUN, IUCS- CESPU), Gandra, Portugal

**Keywords:** Ultrasound, Shear-wave elastography, Septic shock, Intensive care, Muscle wasting, Icu-acquired weakness

## Abstract

**Background:**

Septic shock induces skeletal muscle wasting and neuromuscular dysfunction, contributing to ICU-acquired weakness (ICUAW) and poor recovery after critical illness. Conventional ultrasound primarily captures structural changes but provides limited insight into early muscle mechanical alterations or their relationships with clinical factors. Shear-wave elastography (SWE) enables non-invasive assessment of muscle stiffness, but its performance in critically ill patients remains poorly understood.

**Methods:**

In this prospective study, adults with septic shock requiring invasive ventilation underwent daily muscle ultrasound during the first five ICU days. *Rectus femoris* muscle cross-sectional area, echogenicity and SWE-derived shear modulus (SM_RF_) were assessed bilaterally using conventional ultrasound and shear-wave elastography in both longitudinal and transverse orientations at two anatomical sites within the *rectus femoris*. An exploratory anisotropy estimate was derived from the transverse-to-longitudinal stiffness ratio. ICUAW was evaluated at the time of extubation. Associations between ultrasound markers and clinical variables were explored using correlation and regression analyses.

**Results:**

Twenty-seven patients (66.7% male, mean age 61 ± 12 years) were analysed (125 sessions, 4,971 images). *Rectus femoris* cross-sectional area declined from 3.90 cm² on Day 1 to 2.97 cm² by Day 5 (− 21%, *p* < 0.01), while echogenicity remained stable. Shear modulus (SM_RF_) demonstrated region- and orientation-dependent behaviour. At the lower-third region, longitudinal SM_RF_ remained relatively stable (7.08 to 7.72 kPa), whereas transverse SM_RF_ declined significantly (15.20 to 12.30 kPa, *p* = 0.009), resulting in dynamic changes in the transverse-to-longitudinal stiffness ratio. Muscle atrophy trajectories were similar in patients with and without ICUAW, and no significant between-group differences in SM_RF_ measures were identified. Higher illness severity (SOFA score, β = 18.18, *p* = 0.003) and cumulative fluid balance (β = 3.32, *p* = 0.002) were associated with increases in longitudinal SM_RF_. Greater early caloric intake (β = 0.076, *p* = 0.006) and increases in echogenicity (β = −0.91, *p* = 0.027) were associated with changes in transverse SM_RF_.

**Conclusions:**

Rapid muscle loss during early septic shock was accompanied by distinct, site- and direction-specific stiffness changes. Dual-plane SWE identified early mechanical changes, although these measures did not discriminate ICUAW status in this cohort. Associations with clinical variables suggest that SWE may help capture early physiological muscle changes in critical illness.

**Supplementary Information:**

The online version contains supplementary material available at https://doi.org/10.1186/s40635-026-00968-4.

## Introduction

Critically ill patients experience rapid muscle wasting during the early phase of intensive care unit (ICU) stay [1]. This process is multifactorial, driven by immobility, sepsis, systemic inflammation, multiorgan failure, and treatments such as corticosteroids or neuromuscular blockers [2]. Low muscle mass early in ICU stay is independently associated with mortality in ventilated patients [3], and ultrasound-derived muscle quality parameters have been linked to disability at hospital discharge [4]. These impairments can persist for years after ICU discharge [5, 6].

Ultrasound offers a bedside, repeatable, non-ionising and non-invasive method to assess muscle quantity and quality in critically ill patients, independent of their ability to cooperate [7–9], allowing evaluation during the very early phases of critical illness. The rectus femoris is among the most frequently investigated muscles in intensive care [7, 9–13]; its superficial location and bipennate architecture make it particularly well suited for ultrasound evaluation of muscle structure and mechanical properties. Among emerging ultrasound techniques, shear-wave elastography (SWE) enables real-time estimation of muscle stiffness, providing insight into tissue mechanical properties and integrity. However, most ultrasound studies in critically ill patients have primarily focused on muscle size or echogenicity, while the early evolution of muscle mechanical properties assessed by SWE remain largely unexplored. Existing studies have reported inconsistent SWE methodologies and findings [14–19].

Muscle architecture, including differences in fibre orientation and connective tissue composition, varies between muscles and within different regions of the same muscle. These structural variations can influence the propagation of shear waves and therefore the estimated shear modulus. In addition, skeletal muscle exhibits anisotropy, meaning that its mechanical behaviour differs depending on the direction of measurement relative to muscle fibres (e.g., transverse vs. longitudinal) [20–22]. Changes in anisotropy may therefore provide insights into microstructural alterations occurring during acute muscle injury or disuse. Consequently, distinct regions of the same muscle may respond differently to acute physiological stress. A recent ICU study incorporated SWE into multimodal ultrasound protocols to explore skeletal muscle mechanical properties during critical illness, but the physiological interpretation of stiffness changes and the influence of probe orientation remain poorly understood, especially as it relied on only two distant time points [18]. The first days of septic shock represent a critical phase characterised by intense systemic inflammation, metabolic stress, and profound immobilisation, conditions that may trigger early alterations in skeletal muscle structure and mechanical properties. Yet, longitudinal data specifically describing the early trajectory of muscle mechanical properties during this acute phase in this population has not yet been presented.

The present clinical mechanistic study aimed to characterise the early evolution of *rectus femoris* muscle integrity and its relationship with ICU-acquired weakness in patients with septic shock, using ultrasound markers of muscle structure and mechanical properties, including cross-sectional area (CSA_RF_), echogenicity (ECHO_RF_), SWE-derived shear modulus (SM_RF_).

## Methods

### Study design and population

This prospective investigation was part of the MUSiShock research project [23], an observational study on muscle alterations in septic shock. It was conducted in a 24-bed mixed medical-surgical ICU at a Swiss university hospital (October 2020–August 2024). Recruitment was intermittently interrupted due to COVID-19-related ICU strain and staff reallocation. Ethical approval was granted by the local research ethics board (*Commission Cantonale d’Éthique de la Recherche*, protocol 2020–00452, approved July 28, 2020), and the study was registered on ClinicalTrials.gov (NCT04550143). Procedures followed the Declaration of Helsinki. Under Swiss law for research in emergency situations, initial inclusion was authorised by an independent physician, with written informed consent later obtained from patients or legal representatives. This manuscript follows the STROBE (Strengthening the Reporting of Observational Studies in Epidemiology) guidelines [24].

**Fig. 1:**
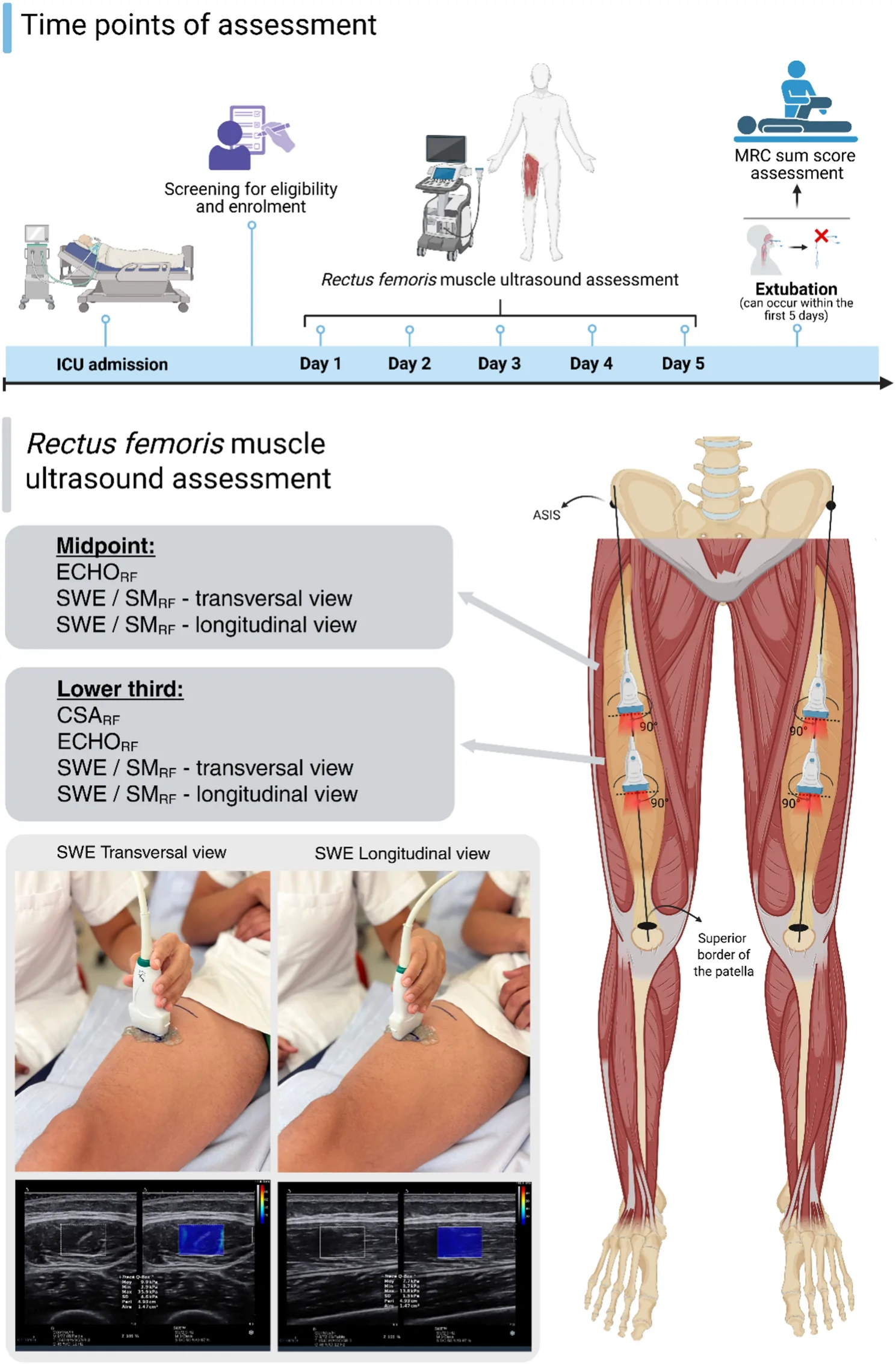
Time points of assessment (upper panel) and ultrasound assessment of the *rectus femoris* muscle (lower panel). The assessment sites (midpoint and lower-third) were identified by measuring the distance between the ASIS and the superior border of the patella. The lower left quadrant illustrates probe placement over the skin in both transverse and longitudinal orientations, with corresponding SWE images. ASIS – Anterior Superior Iliac Spine; CSA_RF_ – Cross-Sectional Area of the r*ectus femoris* muscle; ECHO_RF_ – Echogenicity of the r*ectus femoris* muscle; MRC – Medical Research Council; SM_RF_ – Shear Modulus of the *rectus femoris* muscle; SWE – Shear-Wave Elastography. Image created with BioRender.com

Screening occurred within 24 h of ICU admission (working days). Inclusion criteria were age ≥ 18 years, septic shock (Sepsis-3) [25, 26], Sequential Organ Failure assessment (SOFA) score ≥ 8, lactate > 2 mmol/L, and an anticipated need for > 48 h of mechanical ventilation and ≥ 5 ICU days. Patients had to be ambulatory before admission (walking aids allowed). Exclusion criteria included pregnancy; major lower limb or thoracic injuries; neuromuscular or central nervous system diseases; spinal cord injury; inter-ICU transfer; diaphragm pacemaker; palliative care; sarcopenia, cachexia, or anorexic disorders (per international definitions [27]); and cognitive impairment precluding cooperation. Some criteria reflected broader project requirements. Once enrolled, participants underwent bilateral *rectus femoris* muscle ultrasound on the day of inclusion, followed by daily assessments through day five. The Medical Research Council (MRC) sum score was assessed at extubation (Fig. 1).

### Study procedures

#### Ultrasound assessments

Ultrasound measurements were conducted using a B-mode system with Shear Wave Elastography (SWE) capabilities (Aixplorer^®^, SuperSonic Imagine™) and a 10–2 MHz linear probe. The *rectus femoris* muscle was assessed bilaterally at two standardised anatomical sites: the midpoint and the distal third of the line between the anterior superior iliac spine and the patella. A detailed description of the ultrasound acquisition and analysis protocol is provided in the Supplementary material.

Patients were assessed in a semi-recumbent position (30–45° elevation), with legs extended and relaxed. Whenever feasible, ultrasound examinations were scheduled at comparable times of day. To minimise transient physiological influences on muscle mechanical measurements, patients were left undisturbed for approximately 15 min before image acquisition. Ample gel and minimal probe pressure were used to avoid compression artefacts. Probe positions were marked on the skin to ensure reproducibility.

All ultrasound procedures, including bedside acquisition and subsequent offline image analysis, were performed by the same investigator (INS), who had more than five years of experience in muscle ultrasound. Offline analyses were conducted according to a predefined and standardised protocol after completion of all ultrasound acquisitions, in order to minimise potential bias related to timepoint interpretation.

CSA_RF_ was assessed in the transverse plane using B-mode imaging. The *rectus femoris* muscle was identified between the superficial and central aponeuroses. Brightness and depth settings were initially adjusted to optimise visualisation of the *rectus femoris* boundaries for CSA_RF_ acquisition. Two real-time clips of 10 s were recorded at bedside, and still images with the clearest contours were analysed offline. The muscle boundary was manually traced beneath the aponeurosis, and surface area was calculated (cm²). Because the proximal site exceeded the probe width in > 85% of patients (Table E1), only the lower-third region was analysed. Three images per site were averaged for analysis.

ECHO_RF_ was assessed from three transverse B-mode images acquired along the CSA_RF_. Ultrasound settings (e.g. gain/brightness and focus) were standardised at the initial assessment and maintained across subsequent time points. A region of interest (ROI, 2 × 2 cm, or the largest square within anatomical limits) was placed within the muscle, avoiding overlap across replicates. Quantitative greyscale analysis was performed in ImageJ (National Institutes of Health, USA) [8, 28], and the mean pixel intensity (0–255) three images was averaged to yield one ECHO_RF_ value per site and time point.

SM_RF_ was evaluated using SWE in both transverse and longitudinal orientations [20–22], perpendicular and parallel to the muscle fibres, respectively (SM_RF−TRANSV_ and SM_RF−LONG_). This process begins by measuring the velocity of the induced shear waves (in m/s), which is then used to calculate the shear modulus using the formula µ = ρ·V², where ρ denotes the assumed tissue density (1,000 kg/m³ in human soft tissue). The resulting stiffness values were displayed in kilopascals (kPa) through a colour-coded elastogram within a predefined ROI. For each site and orientation, at least two 10-second clips were recorded after elastogram stabilisation. Following transverse imaging, the probe was rotated 90° to obtain longitudinal views. Still images were extracted and analysed offline using the “Q-Box Trace” tool, selecting the largest elastogram area and excluding the first second of acquisition. When the elastogram did not fully occupy the ROI, contours were manually delineated. The three images with the largest elastogram surface were averaged to yield one representative SM_RF_ value per site and orientation.

#### Medical research council (MRC) score for ICU-acquired weakness (ICUAW)

Volitional muscle strength was assessed using the MRC sum score whenever patients were able to collaborate. However, because many patients remained deeply sedated or unresponsive during the early ICU course, strength testing was often not feasible. For consistency across participants, ICUAW classification in the present analysis was therefore based on the MRC assessment performed at the time of extubation, representing the earliest time point at which reliable and comparable evaluations could be obtained. Patients without an available MRC assessment at extubation were retained in the overall ultrasound analyses but excluded from ICUAW subgroup analyses.

The MRC sum score evaluates six bilateral muscle groups, with a maximum total of 60 [29]. A score < 48 out of 60 was considered consistent with ICUAW [30, 31]. The MRC score has demonstrated excellent inter-rater reliability (ICC > 0.94) [31, 32]. MRC sum score assessments were performed by physiotherapists from the ICU team who routinely conduct MRC evaluations as part of standard clinical care and are experienced in the administration of this test.

#### Clinical and digital data collection

Clinical data, including demographics, illness severity scores, laboratory variables, nutritional intake, cumulative fluid balance and relevant ICU treatments, were retrieved from the hospital’s electronic medical record systems (Centricity Critical Care Clinisoft^®^, General Electric, Helsinki, Finland, and Dossier Patient Informatisé). Study data were recorded in an electronic Case Report Form using REDCap^®^ (Research Electronic Data Capture), a secure web-based platform hosted by the Hôpitaux Universitaires de Genève) [33, 34]. Additional details are provided in the Supplementary material.

### Statistical analysis

Descriptive statistics summarised patient characteristics and ultrasound-derived measurements. Continuous variables were reported as mean with standard deviation (SD) or median with interquartile range (IQR), as appropriate; categorical variables were reported as n (%). To characterise muscle changes, values of CSA_RF_, ECHO_RF_, and SM_RF_ were first averaged between limbs and then analysed per region (midpoint and lower third) and time point. Day 1 was considered the reference time point for all temporal comparisons and percentage-change calculations. A transverse-to-longitudinal SM_RF_ ratio was calculated as an exploratory anisotropy estimate (SM_RF−TRANSV/LONG RATIO_), based on the direction-dependent behaviour of shear-wave propagation in skeletal muscle [21, 22]. Intra-session reliability was assessed using intraclass correlation coefficients (ICC(A,3) two-way mixed effects, average-measures with 95% confidence intervals, CI). Temporal changes in CSA_RF_, ECHO_RF_ and SM_RF_ were analysed using Wilcoxon signed-rank tests (each day vs. Day 1). Because the distributional assumptions of parametric models were not met for a subset of the outcomes, and in order to apply a single assumption-lean approach consistently across all metrics, temporal changes were assessed with rank-based tests. As a sensitivity analysis, the longitudinal outcomes were additionally modelled with linear mixed-effects models, reported in the supplementary material. Concordance between SM_RF_ views was assessed using the concordance correlation coefficient (CCC, 95% CI), and associations between ultrasound parameters were assessed using Kendall’s tau (τ); both analyses used averaged values from repeated measurements for each patient, day, anatomical site and orientation, as applicable. Participants were stratified by ICUAW at extubation (MRC sum score < 48), and groups were compared using the Wilcoxon rank-sum test.

To explore clinical variables associated with early changes in ultrasound-derived markers, exploratory univariate linear regressions were performed using changes in CSA_RF_ and ECHO_RF_ between Day 1 and Day 5, and in SM_RF_ (longitudinal and transverse views), between Day 1 and Day 3, as the number of available observations at ay 5 recurrently fell below 20, precluding reliable model estimation. Candidate predictors were selected a priori based on clinical plausibility and retained when missing data were < 20%. Variables associated at *p* < 0.10 entered exploratory multivariate models, combining predictors in pairs to limit overfitting, following the rule of ≥ 10 observations per parameter. Regression analyses were considered exploratory and hypothesis-generating.

All tests were two-tailed, with *p* < 0.05 considered statistically significant; no adjustment for multiplicity was applied. Analyses were conducted using R software (v4.2.2, www.r-project.org) and IBM SPSS Statistics (Version 28.0.0.0, IBM Corp., Armonk, NY, USA). Additional details are provided in the Supplementary material.

## Results

During the study period, 250 ICU patients with septic shock were screened: 30 met eligibility criteria; 3 were excluded due to lack of consent, yielding a final sample of 27 patients (Figure E1). Twenty-five patients underwent extubation during the ICU stay. MRC assessment at first extubation was available in 22 patients, of whom 12 were classified as having ICUAW and 10 as not having ICUAW. Among the two patients who were not extubated, one died before extubation and one underwent direct tracheostomy without a preceding extubation attempt. Demographic and clinical characteristics are detailed in Tables 1 and 2, with additional data in Tables E2–E3. Across 125 ultrasound sessions, a total of 4,971 images were included in the analysis (Table E1). Six participants missed at least one complete assessment due to clinical procedures, prone positioning, death, or early ICU discharge.

CSA_RF_ demonstrated excellent intra-session reliability over five days (ICC > 0.992; Tables E4–E5). SM_RF_ measurements showed good-to-excellent reliability (Tables E6–E7), consistent across regions and probe orientations.

### Early changes of muscle ultrasound parameters

**Fig. 2:**
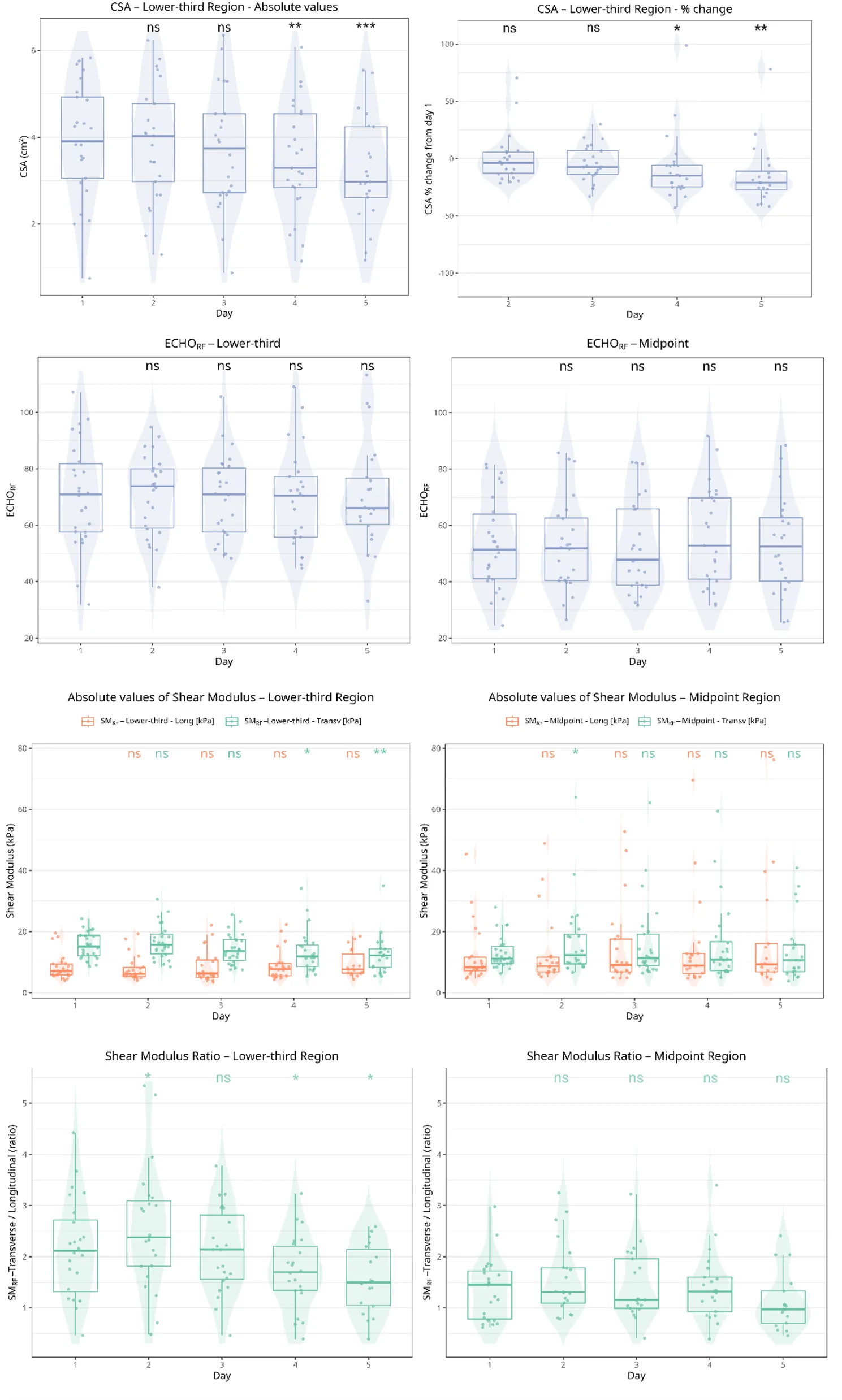
Early evolution of *rectus femoris* ultrasound metrics. Panels are arranged in four rows: the top row shows CSA_RF_ with absolute values on the left and percentage change from day 1 on the right; the second row shows ECHO_RF_ with the lower-third region on the left and the midpoint on the right; the third row presents SM_RF_ for the lower third (left) and midpoint (right) in both longitudinal and transverse views; and the bottom row displays the SM_RF_ transverse-to-longitudinal ratio with the lower third on the left and the midpoint on the right. Violin plots show the distribution (kernel density) with inset boxplots (median, interquartile range, and range), and individual data points are overlaid. Significance: *** *p* ≤ 0.001, ** *p* ≤ 0.01, * *p* ≤ 0.05, ns = not significant. CSA_RF_, Cross-Sectional Area of the *rectus femoris* muscle; ECHO_RF_, Echogenicity of the *rectus femoris* muscle; SM_RF_, Shear Modulus of the *rectus femoris* muscle

The trajectories of CSA_RF_, ECHO_RF_ and SM_RF_ are shown in Fig. 2 and detailed in Tables E8–E10. CSA_RF_ was reported only for the lower-third region, whereas ECHO_RF_ and SM_RF_ were analysed at both anatomical sites.

At the lower-third region, CSA_RF_ declined progressively from Day 1 (median 3.90 cm² [IQR, 3.06–4.92]) to Day 4 (3.29 [IQR, 2.84–4.54], *p* = 0.002) and Day 5 (2.97 [IQR, 2.61–4.24], *p* < 0.001), corresponding to a cumulative reduction of -21.10% [IQR, -27.5 to -10.9]. ECHO_RF_ remained stable (Day 1: 70.9 greyscale units [IQR, 57.6–81.8] vs. Day 5: 66.1 [IQR, 60.2–76.6], *p* = 0.651). In the same region, SM_RF−LONG_ exhibited a mild but non-significant increase (7.08 kPa [IQR, 5.92–9.44] to 7.72 [IQR, 6.43–12.7], + 9.0%), whereas SM_RF−TRANSV_ followed a biphasic trajectory, rising modestly on Day 2 (15.70 kPa [IQR, 12.80–19.20], + 3.3%) before a marked decline on Day 4 (11.90 kPa [IQR, 8.62–15.60], − 21.7%, *p* = 0.023) and Day 5 (12.30 kPa [IQR, 8.33–14.40], − 19.1%, *p* = 0.009). These divergent changes were reflected in the SM_RF−TRANSV/LONG RATIO_, used as an exploratory anisotropy estimate, which initially increased (Day 2: 2.38 [IQR, 1.82–3.09], *p* = 0.012) and subsequently decreased significantly by Day 4 (1.70 [IQR, 1.34–2.21], *p* = 0.010) and Day 5 (1.50 [IQR, 1.05–2.15], *p* = 0.019).

At the midpoint region, ECHO_RF_ also showed no significant variation (51.4 greyscale units [IQR, 41.1–64.0] vs. 52.5 [IQR, 40.2–62.7], *p* = 0.808). SM_RF−LONG_ increased slightly (8.37 kPa [IQR, 7.23–11.7] to 9.30 [IQR, 6.78–16.10], + 11.1%), though not significantly, while SM_RF−TRANSV_ displayed a transient peak on Day 2 (12.3 kPa [IQR, 9.47–19.10], + 9.8%, *p* = 0.041) followed by a progressive decline toward Day 1 values (Day 5: 10.7 kPa [IQR, 6.92–15.70], *p* = 0.926). The SM_RF−TRANSV/LONG RATIO_ exhibited variable day-to-day behaviour (Day 1: 1.45 [IQR, 0.78–1.72] vs. Day 5: 0.97 [IQR, 0.70–1.33], *p* = 0.508), indicating site-specific mechanical heterogeneity without a clear temporal pattern.

### Concordance between probe orientations and correlations between ultrasound parameters

Agreement between SM_RF−LONG_ and SM_RF−TRANSV_ varied by anatomical site (Figure E2). Concordance, expressed as the concordance correlation coefficient (CCC), was good at the midpoint (CCC = 0.76 [95% CI, 0.69–0.81]) and poor at the lower third (CCC = 0.17 [95% CI, 0.08–0.25]).

Overall associations between CSA_RF_, ECHO_RF_ and SM_RF_ are summarised in Table 3 and illustrated in Figure E3. CSA_RF_ showed moderate inverse correlation with SM_RF−LONG_ at both landmarks, and moderate positive correlation with the SM_RF−TRANSV/LONG RATIO_ at lower third point. ECHO_RF_ displayed weak or opposite associations with SM_RF_ depending on probe orientation and anatomical site. Additional correlation data by time are provided in the Supplementary material (Table E11).

### SM_RF_, CSA_RF_ and ECHO_RF_ in relation to ICUAW

**Fig. 3:**
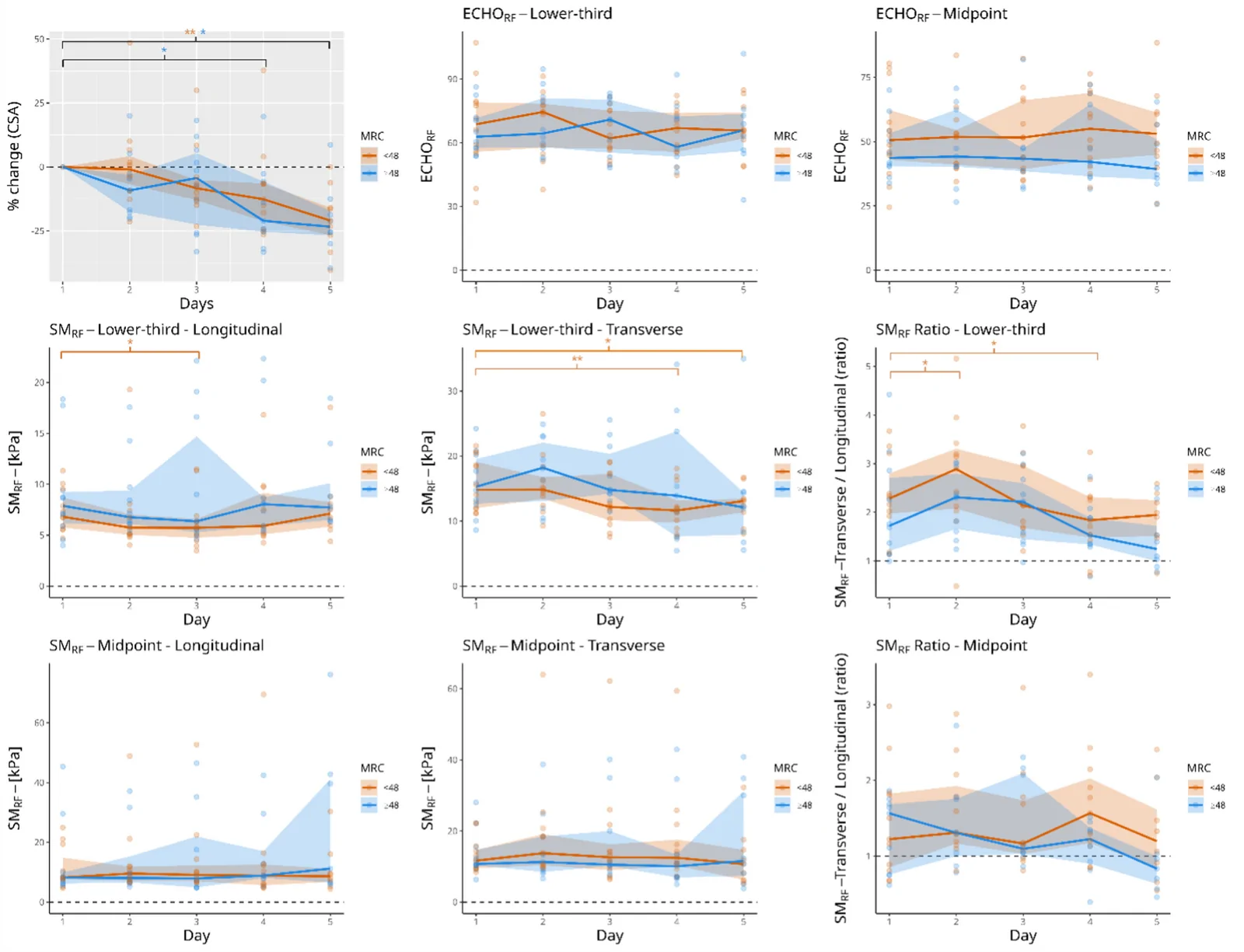
Early trajectories of *rectus femoris* ultrasound metrics, stratified by ICUAW status at first extubation (ICUAW: MRC < 48, *n* = 12, orange; No ICUAW: MRC ≥ 48, *n* = 10, blue). Panels are arranged in a 3 × 3 grid: top row - percentage change in CSA_RF_ at the lower third (left), ECHO_RF_ at the lower third (centre), and ECHO_RF_ at the midpoint (right); middle row (lower-third region) - SM_RF_ in the longitudinal view (left), SM_RF_ in the transverse view (centre), and the SM_RF_ transverse-to-longitudinal ratio (right); bottom row (midpoint region) - SM_RF_ in the longitudinal view (left), SM_RF_ in the transverse view (centre), and the SM_RF_ transverse-to-longitudinal ratio (right). Curves display medians (solid or dashed central lines) with interquartile ranges (shaded areas). Within-group comparisons versus Day 1: * *p* < 0.05; ** *p* < 0.01. No between-group differences were observed at any time point. CSA_RF_, cross-sectional area of the *rectus femoris*; ECHO_RF_, echogenicity of the rectus femoris; SM_RF_, shear modulus of the *rectus femoris*; ICUAW, ICU-acquired weakness; IQR, interquartile range; MRC, Medical Research Council

The evolution of CSA_RF_, ECHO_RF_, and SM_RF_ according to ICUAW status at first extubation is illustrated in Fig. 3.

At the lower-third region, CSA_RF_ declined significantly in both groups (ICUAW: −21.0% [IQR, -26.7 to -15.9]; non-ICUAW: −23.4% [IQR, -26.7 to -17.2] ; *p* < 0.05), without between-group differences. ECHO_RF_ remained stable (ICUAW: 68.7 greyscale units [IQR, 55.8–79.0] to 65.7 [IQR, 62.1–74.3], − 4.4%, *p* = 0.557; non-ICUAW: 62.9 [IQR, 57.6–71.7] to 66.0 [IQR, 56.2–73.6], + 4.9%, *p* = 0.742).

SM_RF−LONG_ decreased significantly in ICUAW by Day 3 (6.82 kPa [IQR, 5.76–8.72] to 5.73 [IQR, 4.75–6.59], − 16.0%, *p* = 0.032) but remained unchanged in non-ICUAW (7.89 kPa [IQR, 6.16–9.23] to 7.72 [IQR, 6.50–10.10], − 2.2%, *p* = 1.000). SM_RF−TRANSV_ declined in ICUAW by Day 4 (14.80 kPa [IQR, 12.00–19.10] to 11.60 [IQR, 9.81–13.20], − 21.6%, *p* = 0.007) and Day 5 (13.10 kPa [IQR, 11.40–13.40], − 11.5%, *p* = 0.039), whereas changes were not significant in non-ICUAW (15.30 kPa [IQR, 12.70–19.50] to 12.10 [IQR, 7.93–14.40], − 21.0%, *p* = 0.195). However, between-group differences in SM_RF−LONG_ and SM_RF−TRANSV_ were not significant at any time point. The SM_RF−TRANSV/LONG RATIO_ was higher in the ICUAW group (Day 1 ratio: 2.28 [IQR, 1.97–2.8]; Day 5: 1.95 [IQR, 1.51–2.25], − 14.5%), with transient variation (Day 2, + 26.8%, *p* = 0.042; Day 4, − 19.3%, *p* = 0.032), though between-group differences were non-significant.

At the midpoint region, ECHO_RF_ remained stable, while SM_RF_ showed limited within-group changes. ECHO_RF_ values were consistently higher in ICUAW, although differences did not reach statistical significance. Additional temporal data are presented in Tables E12–E14 (Supplementary material).

### Exploratory clinical correlates of early changes in *rectus femoris* ultrasound parameters

In univariate analyses (Table 4), higher mean caloric intake during the first three ICU days was associated with greater percentage changes in ECHO_RF_ and SM_RF_, particularly in the transverse orientation. Higher C-reactive protein (CRP) levels were also associated with greater changes in SM_RF−TRANSV_ values, while higher baseline severity, reflected by Sequential Organ Failure Assessment (SOFA) scores, was associated with percentage changes in SM_RF−LONG_, with the corresponding model showing an R^2^ of approximately 0.44. Similarly, a more positive cumulative fluid balance at day 3 was associated with percentage changes in SM_RF−LONG_, with an R² of approximately 0.41. Higher body weight at admission and deeper sedation, as indicated by lower Sedation–Agitation Scale (SAS) scores, were both associated with greater percentage changes in SM_RF−TRANSV_.

In multivariate models (Table 4), combinations of caloric intake with either CRP or ECHO_RF_ changes were associated with SM_RF−TRANSV_ alterations, with model R² values of up to approximately 0.52, whereas models combining body weight and SAS showed R² values of approximately 0.34. Detailed regression coefficients, model sample sizes, confidence intervals, and diagnostic analyses are presented in the Supplementary material (Tables E15–E16).

**Table 1:** Patient demographics and critical illness data

Participants, *n*	27
Male, n (%)	18 (66.7)
Age, years	61.1 (±11.9)
BMI, kg/m^2^	28.7 (±5.3)
SAPS score (0-163) at admission, points	74 [71.2 - 84]
APACHE II (0-71) at admission, points	35 [31.8 - 37]
SOFA score (0-24) at admission, points	11 [10 - 12]
ICU length of stay, days	11 [6.5 - 15.5]
Time to first Ultrasound assessment from ICU admission, hours	16.9 (±5.5)
ICU mortality, n (%)	2 (7.4)
ICU mortality within the first five days, n (%)	1 (3.7)
SAS scale (1-7), averaged value for the first 3 time-points of assessment	1.8 [1.3 - 2.3]
NMBA at least once during the first 5 days, n (%)	21 (77.8)
NMBA average amount for the first 3 days, mg	75.1 (±89.8)
Cumulative fluid balance at day 3, L	7.6 (±5.1)
CRRT, n (%)	4 (14.8)
ECMO, n (%)	3 (11.1)
**Invasive Mechanical ventilation**	
Full support ventilation at day 1, n (%)	20 (74.1)
Partial support ventilation at day 3, n (%)	15 (55.5)
Extubated, n (%)	25 (92.6)
Days to first extubation, days	7.9 [4.6 – 11.0]
**Blood tests**	
PaO_2_/FiO_2_, average of lowest values for each of the first 3 days, kPa	24 (±8.4)
Lactate, average of highest values for each of the first 3 days, mmol/L	3.9 (±3.7)
CRP, average of highest values for each of the first 2 days, mg/L	279.5 (±121.5)
PCT, highest value in the first 2 days, mg/L	42 (±43.4)
Glycemia, average of highest values for each of the first 3 days, mmol/L	9.6 (±2.3)
Leucocyte count, average of highest values for each of the first 3 days, g/L	15 (±6.3)

Values presented as n (%), mean (SD) or median [IQR] as appropriate. IQR values are reported as Q1 to Q3

APACHE II, Acute Physiology and Chronic Health Evaluation II; BMI, Body Mass Index; CRP, C-Reactive Protein; CRRT, Continuous Renal Replacement Therapy; ECMO, Extracorporeal Membrane Oxygenation; FiO₂, Fraction of Inspired Oxygen; ICU, Intensive Care Unit; IQR, interquartile range; N/a, Missing Values; NMBA, Neuromuscular Blocking Agents; PaO₂, Arterial Oxygen Partial Pressure; PCT, Procalcitonin; SAPS, Simplified Acute Physiology Score; SAS, Sedation-Agitation Scale; SD, standard deviation; SOFA, Sequential Organ Failure Assessment; VV-ECMO, Veno-Venous Extracorporeal Membrane Oxygenation; VA-ECMO, Veno-Arterial Extracorporeal Membrane Oxygenation

**Table 2:** – Data related to mobility and nutrition

**Mobility**	
Active mobilisation initiated within the first 5 ICU days, n (%)	11 (40.7)
MRC sum score assessed within the first 5 days, n (%)	7 (25.9)
MRC sum score at first extubation, n (%)	
MRC sum score available at first extubation	22/27 (81.5)
< 48 (ICUAW)	12/27 (44.4)
≥ 48 (no ICUAW)	10/27 (37.0)
Not able to completely follow commands (S5Q) at first extubation	3/27 (11.1)
MRC sum score at first extubation, points	
Overall available patients (*n* = 22)	45.0 [34.0 – 50.8]
< 48 (ICUAW group, *n* = 12)	34.0 [30.0 – 41.5]
≥ 48 (no ICUAW group, *n* = 10)	51.5 [48.5 – 58.0]
Highest IMS score obtained during the first 5 days, points	0.0 [0.0 – 1.5]
IMS (0-10) at first extubation, n (%)	
Level 0 - Nothing (lying in bed)	2 (7.4)
Level 1 - Sitting in bed, exercises in bed	8 (29.6)
Level 2 - Passively moved to chair (no standing)	4 (14.8)
Level 3 - Sitting over edge of bed	4 (14.8)
Level 5 - Transferring bed to chair	5 (18.5)
Level 7 - Walking with assistance of 2 or more people	1 (3.7)
Unknown	1 (3.7)
**Nutrition**	
Feeding start, from ICU admission, days	2.0 [1.5 – 2.0]
Enteral initiated within the first 5 days, n (%)	20 (74.1)
Parenteral initiated within the first 5 days, n (%)	13 (48.1)
Calories, average amount received during the first 3 days, kcal/day	455.3 (±272.1)
Proteins, average amount received during the first 3 days, g/day	9.6 (±9.7)

Values presented as n (%), mean (SD) or median [IQR] as appropriate. IQR is reported as Q1 to Q3. Percentages were calculated using the full cohort (*n* = 27) as the denominator

IQR, interquartile range; ICUAW, ICU-acquired weakness; IMS, ICU Mobility Scale; MRC, Medical Research Council; S5Q, Standardized Five Questions; SD, standard deviation

**Table 3:** Overall correlations between CSA_RF_, ECHO_RF_, SM_RF_ (in both longitudinal and transverse views)

Landmark	Lower third			Midpoint^#^		
pair	Kendall’s tau	*p* value	*n*	Kendall’s tau	*p* value	*n*
CSA_RF_ vs. ECHO_RF_	−0.306 ****	< 0.001	121	−0.362 ****	< 0.001	118
CSA_RF_ vs. SM_RF-LONG_	−0.401 ****	< 0.001	107	−0.250 ***	< 0.001	100
CSA_RF_ vs. SM_RF-TRANSV_	0.098	0.113	119	0.075	0.234	116
CSA_RF_ vs. SM_RF-TRANSV/LONG RATIO_	0.428 ****	< 0.001	107	0.315 ****	< 0.001	100
ECHO_RF_ vs. SM_RF-LONG_	0.264 ****	**< **0.001	110	0.013	0.842	103
ECHO_RF_ vs. SM_RF-TRANSV_	−0.064	0.294	123	−0.219 ****	< 0.001	120
ECHO_RF_ vs. SM_RF-TRANSV/LONG RATIO_	−0.182 **	0.005	110	−0.144 *	0.031	103

CSA_RF_ – Cross-sectional area of the *rectus femoris* muscle (in cm²); ECHO_RF_ – Echogenicity of the *rectus femoris* muscle (greyscale units, 0 to 255); SM_RF-LONG_ – Shear modulus of the *rectus femoris* muscle measured in the longitudinal ultrasound view (in kPa); SM_RF-TRANSV_ – Shear modulus of the *rectus femoris* muscle measured in the transverse ultrasound view (in kPa); SM_RF-TRANSV/LONG RATIO_ – Ratio of Shear modulus values of the *rectus femoris* muscle acquired in the transverse plane relative to the longitudinal plane

^#^CSA_RF_ was only assessed at the lower-third region. Correlations involving midpoint ultrasound parameters therefore used lower-third CSA_RF_ values

* *p* value < 0.05; ** *p* value < 0.01; ***p value < 0.001; ****p value < 0.0001

**Table 4:** Exploratory associations between percentage changes in ultrasound-derived *rectus femoris* parameters and clinical variables (final regression models)

— Univariate models							
**Outcome**	**Time interval**	Predictor	Model *n*	β (SE)	95% CI	***p*** **-value**	**R²**
% Δ ECHO_RF_ (midpoint)	Day 1◊Day 5	Mean caloric intake [kcal/day] (first 3 days)	20	0.030 (0.013)	[0.005, 0.055]	0.032	0.230
% Δ SM_RF-LONG_ (midpoint)	Day 1◊Day 3	SOFA score [points] at admission	19 ^#^	18.178 (4.307)	[10.010, 27.161]	0.003	0.436
% Δ SM_RF-LONG_ (lower third)	Day 1◊Day 3	Cumulative fluid balance at Day 3 [L]	21 ^#^	3.316 (0.992)	[1.124, 4.306]	0.002	0.414
% Δ SM_RF-TRANSV_ (midpoint)	Day 1◊Day 3	Mean caloric intake [kcal/day] (first 3 days)	23 ^#^	0.076 (0.026)	[0.029, 0.126]	0.006	0.252
	Day 1◊Day 3	Mean CRP [mg/L] (first 3 days)	22 ^#2^	0.181 (0.068)	[0.050, 0.335]	0.016	0.217
	Day 1◊Day 3	Δ% ECHO_RF_ (lower third, Day 1–Day 3)	23 ^#^	−0.905 (0.396)	[−1.734, −0.331]	0.027	0.226
% Δ SM_RF-TRANSV_ (lower third)	Day 1◊Day 3	Weight [kg] at admission	24 ^#^	0.986 (0.333)	[0.291, 1.561]	0.006	0.241
	Day 1◊Day 3	SAS [points] (mean first 3 days)	25	15.256 (7.151)	[2.587, 26.874]	0.026	0.137

— Multivariate models							
Outcome	Time interval	Predictors	Model *n*	β (SE)	95% CI	***p*** **-value**	**R²**
% Δ SM_RF-TRANSV_ (midpoint)	Day 1◊Day 3	Mean caloric intake [kcal/day] (first 3 days); CRP [mg/L] (first 3 days)	23 ^#2^	0.074 (0.024); 0.158 (0.050)	[0.024, 0.123]; [0.052, 0.263]	0.001	0.497
% Δ SM_RF-TRANSV_ (midpoint)	Day 1◊Day 3	Mean caloric intake [kcal/day] (first 3 days); Δ% ECHO_RF_ (Day 1–Day 3, midpoint)	23 ^#^	0.081 (0.024); −0.979 (0.297)	[0.032, 0.131]; [−1.598, −0.359]	< 0.001	0.515
% Δ SM_RF-TRANSV_ (midpoint)	Day 1◊Day 3	CRP [mg/L] (first 3 days); Δ% ECHO_RF_ (Day 1–Day 3, midpoint)	23 ^#2^	0.158 (0.072); −0.716 (0.355)	[0.006, 0.309]; [−1.459, 0.026]	0.015	0.356
% Δ SM_RF-TRANSV_ (lower third)	Day 1◊Day 3	Weight [kg] at admission; SAS [points] (mean first 3 days)	23 ^#2^	0.664 (0.367); 14.612 (6.306)	[−0.100, 1.429]; [1.457, 27.766]	0.015	0.342

Only associations reaching *p* < 0.05 are shown. Candidate predictors tested and those retained for further diagnostic evaluation are detailed in the *Supplementary material* (Table E15)

% Δ, percentage change; β, regression coefficient; CI, confidence interval; CRP, C-reactive protein; CSA_RF_, cross-sectional area of the rectus femoris; ECHORF, echogenicity of the rectus femoris; R², coefficient of determination; SAS, Sedation–Agitation Scale; SE, standard error; SM_RF_, shear modulus of the rectus femoris; SOFA, Sequential Organ Failure Assessment

^#^ after removal of one influential case; ^#2^ after removal of two influential cases

For multivariate models, *p*-values and R² refer to the overall model fit. Additional regression diagnostics, bootstrap analyses, and model assumptions are detailed in the *Supplementary material*

## Discussion

This study provides an early-phase, dual-plane, high-resolution ultrasound characterisation of *rectus femoris* changes in septic shock, examining structural (CSA_RF_), qualitative (ECHO_RF_) and mechanical (SM_RF_) properties over the first five ICU days. Four main findings emerged: (a) rapid and substantial muscle wasting; (b) direction-dependent mechanical behaviour, with longitudinal SM_RF_ remaining relatively stable and transverse SM_RF_ declining over time, resulting in a reduction in the estimated anisotropy ratio; (c) no significant between-group differences in SM_RF_ or anisotropy estimates according to ICUAW status at first extubation, despite some exploratory within-group temporal changes; and (d) exploratory clinically relevant correlates of early muscle mechanical changes.

### Early muscle wasting

Consistent with the rapid catabolic response to critical illness, we observed a marked decline in CSA_RF_, with a cumulative median reduction of ~ 21%. This magnitude aligns with prior work showing accelerated muscle wasting in multiorgan failure. In the landmark study by *Puthucheary and colleagues*, CSA_RF_ fell by 12.5% at day 7 and 17.7% at day 10, with the greatest losses in septic patients [7]. Importantly, CSA_RF_ has repeatedly been shown to better reflect true muscle loss than thickness [4, 9, 10, 35]. Recent meta-analysis reported an average CSA_RF_ decline of ~ 2% per day in critical illness [1], while studies in septic populations describe losses of 13–23% within the first week [11–13], consistent with our findings.

### Direction-dependent stiffness and anisotropy

Observed changes in SM_RF_ were directionally dependent, with longitudinal and transverse measurements following distinct temporal patterns over time. Longitudinal SM_RF_ values were already elevated at Day 1 compared with values reported in healthy individuals (~ 3–4 kPa in *rectus femoris* and other lower-limb muscles [36]), approaching those seen in neuromuscular disease cohorts [37], but showed no statistically significant change over time. In contrast, transverse SM_RF_ declined significantly, particularly at the lower-third region where connective-tissue interfaces are more prominent. This divergence resulted in a progressive decline in the transverse-to-longitudinal SM_RF_ ratio, used in this study as an exploratory anisotropy estimate. This direction-specific pattern is consistent with the anisotropic mechanical properties of the skeletal muscle [14, 20], where shear-wave propagation depends on fibre orientation and loading conditions. In an ideal isotropic tissue, shear-wave propagation would be expected to be similar across directions, resulting in a transverse-to-longitudinal ratio close to 1. By contrast, the organised fibre structure of skeletal muscle makes stiffness direction-dependent, In line with this concept, a previous SWE study reported lower anisotropy-related ratios in superficial fat-rich regions and higher ratios within muscle regions [22]. Consistent with previous reports, stiffness values in our study were generally higher in the transverse than in the longitudinal plane [14, 22, 38, 39]. Regional variation was also evident. Concordance between longitudinal and transverse SM_RF_ was moderate-to-strong at the midpoint but poor at the lower third. The lower-third region also showed the greatest early changes in transverse SM_RF_, suggesting that site-specific factors may influence sensitivity to early muscle alterations in septic shock.

Evidence on SWE in critically ill patients remains scarce. A recent ICU study incorporating SWE into a multimodal ultrasound protocol also explored *rectus femoris* stiffness (assessed in the transverse plane) during the first week of critical illness but did not observe significant longitudinal changes [18]. Interestingly, the stiffness values reported in that cohort were substantially higher than those observed in our study. Importantly, measurements were obtained at only two time points, which may limit detection of early dynamic alterations in muscle mechanical properties. More recently, a study combining ultrasound and SWE during neuromuscular electrical stimulation reported resting quadriceps stiffness values in the longitudinal view of approximately 13–14 kPa, somewhat higher than the longitudinal SM_RF_ values observed in our cohort at day 1 (median approximately 7–9 kPa) [19]. These differences may partly reflect variations in patient characteristics and measurement conditions. In particular, our cohort included patients high with septic shock and illness severity, and some assessments were performed under neuromuscular blocking agents. Outside the ICU, altered stiffness has been associated with functional decline in chronic obstructive pulmonary disease [40], sarcopenia [41], and diabetes [42], demonstrating added diagnostic value beyond muscle size. Together these findings and our dual-plane regional analysis suggest that SWE may capture early muscle mechanical changes with potential relevance to the trajectory of critical illness.

The relationships between ultrasound markers were weak-to-moderate (CSA_RF_ vs. SM_RF−LONG_) and weaker or inconsistent (CSA_RF_ vs. SM_RF−TRANSV_). These magnitudes indicate that stiffness changes are only partly associated with atrophy as quantified by CSA_RF_. The progressive decline in the anisotropy estimate tracked the CSA_RF_ reduction, suggesting co-variation but not full dependence on structural loss.

### ICU-acquired weakness subgroup analysis

With respect to ICUAW, both groups exhibited similar CSA_RF_ trajectories, consistent with prior findings that early mass loss alone does not discriminate weakness [4, 9, 43, 44]. Some within-group temporal changes in SM_RF_ and the anisotropy estimate were observed at the lower-third region in patients classified as having ICUAW at first extubation. These changes were observed before the extubation-based strength assessment, but did not translate into significant between-group differences, highlighting the complexity of early neuromuscular dysfunction in critical illness. A previous study has suggested that combining SWE with structural markers may contribute to the early identification of ICUAW [17]. In our cohort, however, SWE-derived measures did not discriminate ICUAW status. The observed within-group temporal changes should therefore be interpreted as exploratory findings, suggesting that directional stiffness may capture aspects of muscle behaviour not reflected by CSA_RF_ alone, rather than providing evidence of discriminatory performance. These observations require confirmation in larger cohorts.

### Clinical correlates of muscle mechanical changes

Exploratory associations with early ultrasound changes suggested clinically plausible relationships and were region-specific. Higher SOFA scores were associated with greater increases in longitudinal SM_RF_ at the midpoint, and more positive cumulative fluid balance was associated with higher longitudinal SM_RF_ at the lower third. Conversely, higher early caloric delivery and greater inflammatory burden (CRP) were linked to increases in transverse SM_RF_ at the midpoint, while higher baseline body weight and deeper sedation were associated with greater transverse SM_RF_ increases at the lower third. These findings suggest possible associations between directional stiffness and metabolic, inflammatory and fluid-related stressors in septic shock, but should be interpreted as hypothesis-generating.

The association between positive fluid balance and rising longitudinal stiffness is particularly notable, aligning with the capillary leak physiology of septic and inflammatory shock [45]. SWE may reflect tissue responses to fluid redistribution earlier than structural measures, although this requires mechanistic confirmation. Higher early caloric intake was also linked to increased transverse stiffness; whether this reflects metabolic support or early cellular strain remains uncertain, as current guidance supports early enteral nutrition once shock is controlled, while avoiding rapid full target feeding [46].

Our dense daily assessment may therefore have facilitated detection of early mechanical changes that could remain undetected in studies using more widely spaced time points. These observations suggest that directional stiffness may be associated with early physiological stress responses not reflected by muscle size alone.

### Strengths and limitations

This study provides one of the first longitudinal assessments of muscle structure and mechanics exclusively in septic shock using dual-plane SWE. Strengths include daily bilateral assessments during the early phase of critical illness, rigorous image-acquisition procedures, and high intra-operator reliability, enabling detection of short-term directional changes.

Several limitations merit consideration. The cohort size constrained subgroup analyses, and functional assessment was performed only once. Regression analyses were exploratory and based on a limited effective sample size, with outcomes expressed as percentage change scores. CSA_RF_ analysis was restricted to the lower-third region, limiting structural comparisons between anatomical sites. Ultrasound-based stiffness measurements may be influenced by tissue hydration or body composition. In septic shock, however, these factors may also reflect pathophysiological processes such as fluid redistribution and metabolic stress, which were explored in our regression analyses. SWE can be influenced by involuntary muscle activity, although deep sedation likely minimised this. Finally, anisotropy estimates require external validation.

### Clinical implications and future directions

These findings highlight the potential role of advanced ultrasound techniques in the early assessment of skeletal muscle health in septic shock. Dual-plane SWE, particularly at anatomically heterogeneous regions such as the lower third of the *rectus femoris*, may help capture early muscle mechanical changes when volitional testing is not feasible. Standardisation of SWE protocols, validation of anisotropy indices, and clarification of physiological meaning across disease states are required. Future studies should evaluate associations with early mobilisation, functional recovery, long-term weakness and responses to nutrition and fluid strategies. Larger cohorts and automated dual-direction SWE platforms may enhance feasibility and clinical adoption.

## Conclusion

This study demonstrates rapid and substantial *rectus femoris* wasting during early septic shock, accompanied by direction-dependent alterations in muscle mechanical properties assessed by shear-wave elastography. Longitudinal stiffness remained relatively stable while transverse stiffness declined, resulting in reduced estimated anisotropy and suggesting mechanical information not captured by muscle size alone. SWE-derived measures did not discriminate ICU-acquired weakness status in this cohort, while their associations with clinical variables support further investigation of dual-plane SWE to characterise early skeletal muscle changes in critical illness.

## Supplementary Information

Below is the link to the electronic supplementary material.

## Data Availability

The datasets used and/or analysed during the current study are available from the corresponding author on reasonable request.

## Abbreviations

BCa: Bias-corrected and accelerated
B-mode: Brightness Mode
CI: Confidence interval
CRP: C-reactive protein
CSA_RF_: Cross-sectional area of rectus femoris muscle
ECHO_RF_: Echogenicity of rectus femoris muscle
ICC: Intra-class correlation coefficient
ICU: Intensive care unit
IQR: Interquartile range
ICUAW: ICU-acquired weakness
ROI: Region of interest
SAS: Sedation–agitation scale
SD: Standard deviation
SM_RF_: Shear modulus of rectus femoris muscle
SOFA: Sequential Organ Failure Assessment
SWE: Shear-wave elastography
