# Supplementary material for: Early mechanical changes of limb muscle in septic shock assessed by dual-plane shear-wave elastography
Source: Intensive Care Med Exp. 2026 Sep 2;14:117. doi: 10.1186/s40635-026-00968-4 (PMC13538337; doi:10.1186/s40635-026-00968-4)
Supplement: Supplementary file 1 [file 40635_2026_968_MOESM1_ESM.pdf]

<u>**Supplementary material**</u>

Early mechanical changes of limb muscle in septic shock assessed by dual-plane shear-wave elastography

Ivo **NETO SILVA** [1,2,3,4], Rui **AZEVEDO** [5,6], Joana **DUARTE** [1], Stanislas **ABRARD** [2,7,8,9] , José Alberto **DUARTE** [4,10], Karim **BENDJELID** [1,2]

Affiliations:

[1] Intensive Care Division, Department of Acute Medicine, Geneva University Hospitals, Geneva, Switzerland

[2] Geneva Hemodynamic Research Group, Faculty of Medicine, University of Geneva, Geneva, Switzerland.

[3] Care Directorate, Geneva University Hospitals, Geneva, Switzerland

[4] Centre of Physical Activity, Health and Leisure (CIAFEL), Faculty of Sport, University of Porto, Porto, Portugal.

[5] Associate Laboratory i4HB - Institute for Health and Bioeconomy, University Institute of Health Sciences - CESPU, Gandra, Portugal.

[6] UCIBIO - Applied Molecular Biosciences Unit, Forensic Sciences Research Laboratory, University Institute of Health Sciences (1H-TOXRUN, IUCS-CESPU), Gandra, Portugal.

[7] Anaesthesiology Division, Department of Acute Medicine, Geneva University Hospitals, Geneva, Switzerland.

[8] Department of Anaesthesiology and Critical Care Medicine, Hospices Civils de Lyon, Edouard Herriot Hospital, Lyon, France.

[9] Research on Health care Performance (RESHAPE), INSERM U1290, University Claude Bernard Lyon 1, Lyon, France.

[10] 1H-TOXRUN-Toxicology Research Unit, University Institute of Health Sciences - CESPU, Gandra, Portugal

STUDY PROCEDURES

Ultrasound assessments

Ultrasound images were acquired using a B-Mode system (Aixplorer®, SuperSonic Imagine™, Aix-en-Provence, France) equipped with Shear Wave Elastography (SWE) capabilities and a linear transducer (SL 10-2) operating within a 10–2 MHz frequency range.

The bilateral *rectus femoris* muscle of each participant, which is a bipennate muscle with fibers oriented obliquely on either side of a central aponeurosis [1], was evaluated at two distinct anatomical locations [2]:

- the midpoint between the anterior superior iliac spine and the superior border of the patella (proximal site), and
- the junction between the lower third and upper two-thirds of the same line (distal site).

Participants were positioned semi-recumbently, with the head of the bed elevated between 30° and 45°, and the knees in a passive, extended, and neutral position. In cases where patients were conscious, they were instructed to remain still throughout the procedure. Whenever feasible, ultrasound examinations were performed at comparable times of day. Prior to each assessment, patients were left undisturbed for approximately 15 minutes in order to minimise transient physiological influences on muscle mechanical properties, such as recent mobilisation or clinical interventions.

At both anatomical sites, assessments included the cross-sectional area (CSA_{RF}) and the shear modulus (SM_{RF}) derived via SWE. To minimize tissue compression and avoid artefactual distortion, generous amounts of ultrasound gel were used, and light transducer pressure was applied. Probe positions were marked on the skin to ensure consistent placement across all time points. All ultrasound procedures, both at the bedside and during subsequent offline analysis, were performed by the same investigator (INS), who had over five years of experience in muscle ultrasound. Offline analyses were conducted according to a predefined and standardised protocol after completion of all ultrasound acquisitions, in order to minimise potential bias related to timepoint interpretation.

a) <u>*Rectus femoris* muscle Cross-Sectional Area (CSA_{RF})</u>

For the assessment of CSA_{RF}, an anterior view of the thigh was obtained using Brightness mode (B-Mode) with a musculoskeletal preset (a tissue tuner at 1,540 m/s and dynamic range at 72 dB), and the ultrasound probe was placed in a transverse orientation (perpendicular to the muscle fibres). The *rectus femoris* muscle was identified as the area between the superficial anterior aponeurosis and the central aponeurosis, which borders the vastus intermedius muscle. When the borders were close but not fully visualised, a wider imaging mode (trapezoidal mode) was activated to extend the imaging window. Brightness and depth settings were initially adjusted to optimise visualisation of the rectus femoris boundaries for CSA_{RF} acquisition. A hyperechoic line corresponding to the femur was used as a reference to set the appropriate image depth. Subsequently, two 10-second real-time clips were acquired at the bedside.

From these recordings, frames where muscle boundaries were most clearly defined were selected for analysis. The CSA_{RF} was measured using the standard surface area tool available in the ultrasound system, by manually tracing the area beneath the hyperechoic aponeurosis on a frozen image using the on-screen cursor. Due to limitations in visualising the entire muscle at the proximal site, where the muscle extended beyond the ultrasound field in more than 85% of participants, only the distal landmark was included in the CSA_{RF} analysis. At each assessment, three images with variability <10% were selected, and CSA_{RF} values (in cm^2) were averaged for use in statistical analyses.

b) *Rectus femoris* muscle Echogenicity ($ECHO_{RF}$)

For $ECHO_{RF}$, three images were obtained along the CSA_{RF}. Ultrasound settings (*e.g.* gain/brightness and focus) were standardised at the initial assessment and maintained across subsequent time points. Quantitative greyscale analysis was undertaken using ImageJ software (National Institutes of Health, USA) [3, 4]. For each image, a region of interest (ROI) measuring 2 × 2 cm was first selected whenever feasible; if this was not possible, the largest square ROI within the anatomical boundaries of the muscle was used. The ROI placement was varied across the three images to avoid overlap between assessed regions and to provide a more representative assessment of overall tissue quality. From each ROI, a histogram of pixel intensity values (ranging from 0 to 255, where black = 0 and white = 255) was then generated, and the mean pixel value of the distribution was derived. The mean $ECHO_{RF}$ value from each image was retrieved, and the three values were subsequently averaged to obtain a single $ECHO_{RF}$ measurement per time point for analysis.

c) *Rectus Femoris* muscle Shear Modulus (SM_{RF})

SWE is an ultrasound-based technique that uses an acoustic radiation force, referred to as a "push beam", to create shear waves within biological tissues. The subsequent tissue displacement is captured by the ultrasound system, allowing for the estimation of mechanical properties such as tissue stiffness [5, 6]. This process begins by measuring the velocity of the induced shear waves (V, in metres per second), which is then used to calculate the shear modulus (SM) using the formula $\mu = \rho \cdot V^2$, where ρ denotes the assumed tissue density (1,000 kg/m^3 in human soft tissue). The resulting SM values are expressed in kilopascals (kPa).

Tissue stiffness is visualised using a colour-coded elastogram, which displays the distribution of SM values within a designated region of interest (ROI). In this context, higher SM values correspond to stiffer tissue characteristics. For further technical and biomechanical details related to SWE application in skeletal muscle assessment, readers are referred to comprehensive reviews in the literature [5, 6].

In this study, SM_{RF} was assessed using two probe orientations: transverse (perpendicular to the muscle fibres, as used for CSA_{RF}, referred to as $SM_{RF\text{-}TRANSV}$) and longitudinal (parallel to the fibres, $SM_{RF\text{-}LONG}$), as illustrated in Figures 1 from the manuscript. Following initial identification of the *rectus femoris* muscle, as described for the CSA_{RF} measurements, the ultrasound system was switched to SWE mode. A ROI was defined to encompass the largest possible area of the *rectus femoris* muscle, with the acquisition frequency set at 2 Hz, the maximum supported by the equipment. When this frequency could not be achieved due to muscle depth, the closest possible frequency was selected.

The probe was held in position until the elastogram stabilised and remained steady throughout image acquisition. The transducer was then rotated 90 degrees so that the probe marker faced cranially, and the acquisition process was repeated for the longitudinal orientation. At each anatomical site and for each probe orientation, a minimum of two real-time clips lasting 10 seconds were collected at the bedside, with more acquired when necessary to ensure sufficient image quality and elastogram consistency.

From these pre-acquired SWE clips, still images were extracted and analysed offline. The entire elastogram within the pre-defined ROI was selected using the "Q-Box Trace" function, enabling quantification of stiffness. The first second of each clip was excluded to ensure elastogram stabilisation. In instances where the elastogram did not fully occupy the ROI, the visible elastogram contour was manually delineated. For each selected image, the mean shear modulus and elastogram surface were retrieved (see examples in Figure 1 from the manuscript).

The three images showing the largest elastogram surface area were selected for further analysis, based on the principle that a greater surface coverage indicates better data quality. When multiple images had equal surface areas, a secondary criterion was applied: preference was given to those with a mean shear modulus variability below (or close to) 10%. The average of the three retained images was used for analysis. Results were expressed in kilopascals (kPa).

Medical Research Council (MRC) sum score

Volitional muscle strength was assessed using the MRC sum score whenever patients were able to collaborate. However, because many patients remained deeply sedated or unresponsive during the early ICU course, strength testing was often not feasible. For consistency across participants, ICUAW classification in the present analysis was therefore based on the MRC assessment performed at the time of extubation, representing the earliest time point at which reliable and comparable evaluations could be obtained. MRC sum score assessments were performed by physiotherapists from the ICU team who routinely conduct MRC evaluations as part of standard clinical care and are experienced in the administration of this test. Patients without an available MRC assessment at extubation were retained in the overall ultrasound analyses but excluded from ICUAW subgroup analyses.

To determine whether patients were able to participate in voluntary assessments, the Standardised Five Questions (S5Q) tool was used. This method involves five simple verbal commands: “open and close your eyes”, “look at me”, “open your mouth and stick out your tongue”, “nod your head”, and “raise your eyebrows when I count to five” [7, 8]. A minimum of 3 correct responses out of 5 was required to classify a patient as “able to collaborate”. This threshold has been previously used in studies evaluating ICU-acquired weakness to determine the patient’s ability to participate in volitional strength testing.

Only patients who met this threshold were eligible for manual muscle strength evaluation using the Medical Research Council (MRC) sum score, a tool that requires active patient participation. The MRC score assesses six muscle groups bilaterally: shoulder abduction, elbow flexion, wrist extension, hip flexion, knee extension, and ankle dorsiflexion [9-11]. Each group is graded on a scale from 0 to 5:

0 – no visible or palpable contraction.

1 – contraction without limb movement.

2 – movement not against gravity.

3 – movement against gravity.

4 – movement against gravity with some resistance.

5 – normal strength [10, 12].

The total score ranges from 0 to 60. A score below 48 is generally considered indicative of "significant weakness" [7, 13-16]. The MRC sum score has shown excellent inter-rater reliability, with intraclass correlation coefficients (ICCs) above 0.94 [16, 17]. Values obtained were used for further analysis.

Other clinical data

Clinical data were retrieved from the hospital's electronic medical record systems, including the ICU clinical information system (Centricity Critical Care Clinisoft®, General Electric, Helsinki, Finland) and the hospital electronic patient record (Dossier Patient Informatisé). When applicable, perioperative information was additionally obtained from the anaesthesia information system (MetaVision; iMDSoft®, Tel Aviv, Israel).

Variables collected included demographic data, illness severity scores, laboratory parameters, nutritional intake, cumulative fluid balance, and relevant ICU treatments. Fluid balance was calculated as the cumulative difference between fluid intake and output recorded in the ICU clinical information system up to the time of each ultrasound assessment. When available, perioperative fluid data were retrieved from the anaesthesia information system and hospital electronic medical records. Nutritional intake, including daily caloric delivery, was extracted from the ICU clinical information system and corresponded to the values recorded up to the time of each ultrasound assessment.

When available, data were prospectively integrated during the ICU stay and subsequently verified through systematic extraction from the electronic records. All study variables were entered into an electronic Case Report Form (eCRF) using REDCap® (Research Electronic Data Capture), a secure, web-based platform hosted by the Hôpitaux Universitaires de Genève [18, 19]. REDCap® is specifically designed to support the collection, management, and storage of research data in clinical studies.

Statistics

Descriptive statistics were used to summarise patient characteristics and ultrasound-derived measurements. Continuous variables were reported as mean (standard deviation [SD]), median (interquartile range [IQR]), and absolute and relative frequencies, as appropriate. To characterise muscle changes, CSA_{RF}, $ECHO_{RF}$ and SM_{RF} values from both limbs were averaged per region (midpoint and lower third) and time point. A transverse-to-

longitudinal SM_{RF} ratio was computed by dividing values from the transverse view by those from the longitudinal view, serving as a proxy for the degree of muscle anisotropy [20, 21].

Intra-session reliability was assessed using intraclass correlation coefficients (ICC(A,3) two-way mixed effects, average-measures with 95% confidence intervals, CI), interpreted according to standard thresholds. Temporal changes in CSA_{RF}, $ECHO_{RF}$ and SM_{RF} were analysed using Wilcoxon signed-rank tests (each day vs Day 1). Because the distributional assumptions of parametric models were not met for a subset of the outcomes, and in order to apply a single assumption-lean approach consistently across all metrics, temporal changes were assessed with rank-based tests. As a sensitivity analysis, the longitudinal outcomes were additionally modelled with linear mixed-effects models, reported in the supplementary material. Concordance between ultrasound views was evaluated using the concordance correlation coefficient (CCC, 95% CI), stratified by region. Associations between CSA_{RF}, $ECHO_{RF}$ and SM_{RF} (longitudinal, transverse, and ratio) were examined using Kendall's tau (τ), both overall and by day and region.

For exploratory subgroup analyses, patients were stratified by the presence of ICU-acquired weakness at the time of extubation (MRC sum score <48). Between-group comparisons of values and percentage changes were performed using the Wilcoxon rank-sum test.

To explore clinical variables associated with muscle quantity and quality changes, univariate linear regressions were performed using percentage changes in CSA_{RF} and $ECHO_{RF}$ between Day 1 and Day 5, and in SM_{RF} (longitudinal and transverse views) between Day 1 and Day 3 as dependent variables and selected clinical parameters as predictors. The shorter time window for SM_{RF} was chosen because the number of available observations at day 5 recurrently fell below 20, precluding reliable model estimation. The number of available observations varied across time points due to ICU discharge, death, or missed assessments.

Candidate variables were chosen a priori based on clinical plausibility and included demographic, anthropometric, severity, sedation, neuromuscular blockade, fluid balance, nutritional, inflammatory, metabolic and early ultrasound-derived variables. These variables were included only when less than 20% of data were missing. The full list of candidate predictors considered for exploratory regression analyses is provided in Table E15. Predictors associated with the dependent variable at $p < 0.10$ in these univariate models were retained for further diagnostic evaluation and multivariate exploration.

Each regression model underwent systematic diagnostic evaluation. Residual normality was assessed using the Shapiro–Wilk test, and homoscedasticity was evaluated with the Glejser and White tests. Multicollinearity was examined through tolerance and variance inflation factor (VIF) statistics. Influential observations were identified using Cook's distance and studentised residuals (thresholds: Cook's D > 4/N or |studentised residual| > 3). When assumptions were violated or influential points detected, analyses were repeated after exclusion, and the most robust model—satisfying all assumptions—was retained. Bias-corrected and accelerated (BCa) bootstrap confidence intervals (5,000 resamples) were computed not only when residuals deviated from normality, but also to confirm the robustness and stability of statistically significant results. Quadratic terms were tested when evidence of non-linearity was suggested either by residual diagnostics, model fit indices, or theoretical considerations, and further verified through visual inspection of

partial residual plots. Model fit was evaluated using R^2 and adjusted R^2 values, and ΔR^2 was used to assess the contribution of quadratic terms.

Multivariate linear regressions were subsequently conducted to assess additive effects between independent predictors identified at $p < 0.10$ in univariate analyses. Variables were entered in pairs to minimise overfitting, in accordance with the conventional rule of at least ten observations per estimated parameter to ensure model stability. Model adequacy was verified using the same diagnostic framework (normality, homoscedasticity, and multicollinearity). The Durbin–Watson statistic was applied to assess the independence of residuals in multivariate models only. Final models reported both standardised and unstandardised coefficients, standard errors, 95% confidence intervals, and corresponding p-values, together with diagnostic indices (VIF, tolerance, and residual tests).

All tests were two-tailed, with $p < 0.05$ considered statistically significant; no adjustment for multiplicity was applied. Analyses were conducted using R software (v4.2.2, www.r-project.org) and IBM SPSS Statistics (Version 28.0.0.0, IBM Corp., Armonk, NY, USA).

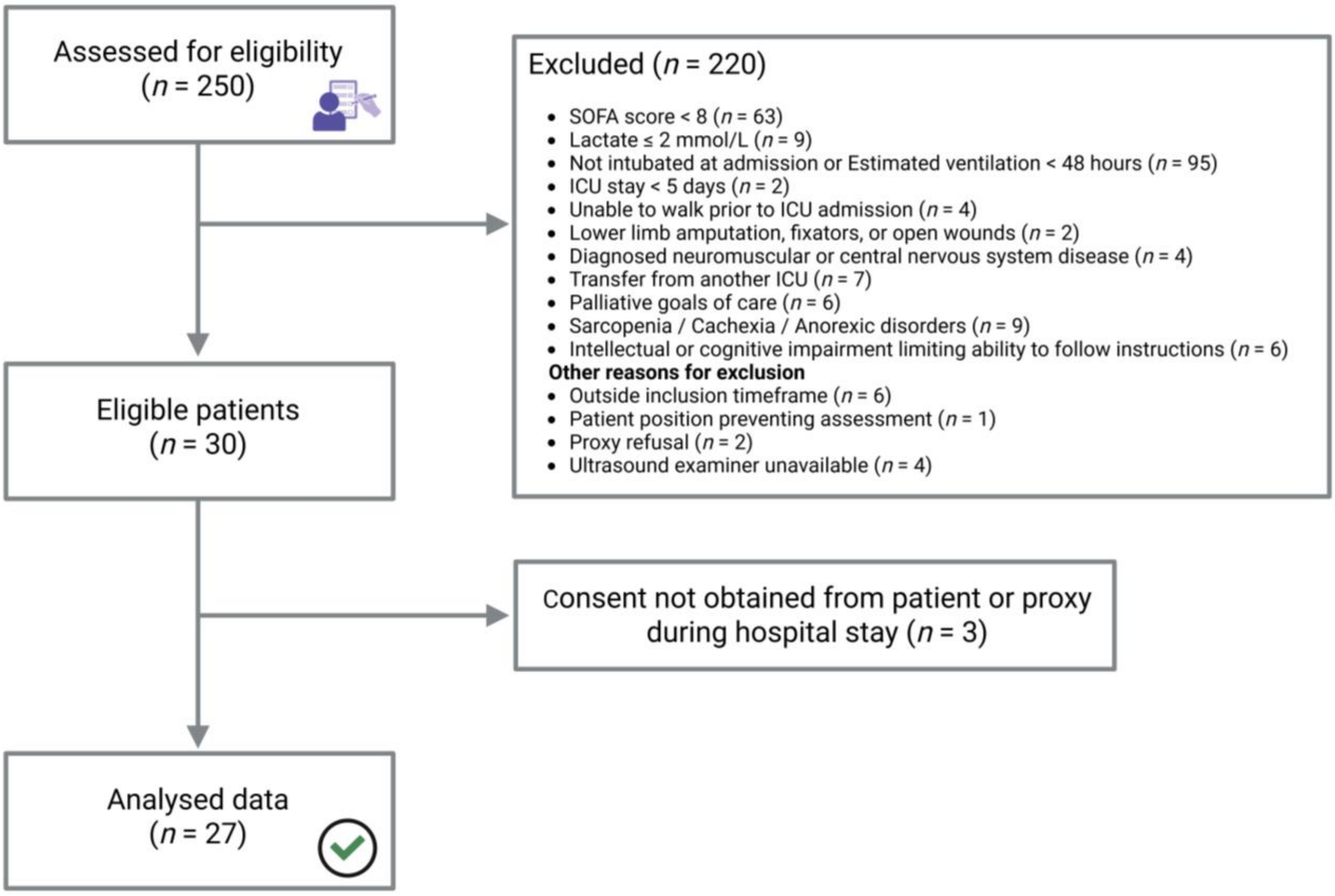

Figure E1 – Flow diagram of participant inclusion and exclusion.

ICU, Intensive Care Unit; SOFA, Sequential Organ Failure Assessment.

**Table E1** – Number of assessments of quadriceps *rectus femoris* muscle.		
**Type of Assessment**	**Total**	**% total**
Daily assessments (overall)	125	100.0
CSA_{RF} – Right side – Midpoint	17	13.6
CSA_{RF} – Right side – Lower third	121	96.8
$ECHO_{RF}$ – Right side – Midpoint	119	95.2
$ECHO_{RF}$ – Right side – Lower third	124	99.2
SWE – Right side – Midpoint – Transverse view	117	93.6
SWE – Right side – Midpoint – Longitudinal view	98	78.4
SWE – Right side – Lower third – Transverse view	122	97.6
SWE – Right side – Lower third – Longitudinal view	108	86.4
CSA_{RF} – Left side – Midpoint	16	12.8
CSA_{RF} - Left side – Lower third	120	96.0
$ECHO_{RF}$ – Left side – Midpoint	121	96.8
$ECHO_{RF}$ – Left side – Lower third	124	99.2
SWE – Left side – Midpoint – Transverse view	119	95.2
SWE – Left side – Midpoint – Longitudinal view	101	80.8
SWE – Left side – Lower third – Transverse view	122	97.6
SWE – Left side – Lower third – Longitudinal view	108	86.4

Values presented in absolute value and percentage of total daily assessments.

CSA_{RF}, Cross-Sectional Area of the r*ectus femoris* muscle; $ECHO_{RF}$ – Cross-Sectional Area of the r*ectus femoris* muscle; SWE, shear-wave elastography.

**Table E2** - Patient demographics and critical illness data (Additional Data Table).		
Variable	***Result***	***N/a***
Admitted from, n (%)		
Emergency department	13 (48.1)	
Operating/Recovery room	7 (25.9)	
Hospital ward	6 (22.2)	
Other Hospital	1 (3.7)	
Frailty scale (1-9), before admission, n (%)		
1 (Very Fit)	1 (3.7)	
2 (Well)	5 (18.5)	
3 (Managing Well)	10 (37.0)	
4 (Vulnerable)	4 (14.8)	
5 (Mildly frail)	4 (14.8)	
6 (Moderately frail)	3 (11.1)	
7 (Severely Frail), 8 (Very Severely Frail), 9 (Terminally Ill)	0 (0)	
Source of Infection, n (%)		
Abdominal	14 (51.9)	
Respiratory	10 (37.0)	
Bloodstream	1 (3.7)	
Other	1 (3.7)	
Unknown	1 (3.7)	
SOFA score – PaO_2/FiO_2 (0-4)	3 [2.5 - 3]	
SOFA score – Platelets (0-4)	0 [0 – 2]	
SOFA score – Glasgow Coma Score (0-4)	0 [0 - 1]	
SOFA score – Bilirubin (0-4)	1 [0 - 2]	
SOFA score – MAP or vasoactive agents (0-4)	4 [4 - 4]	
SOFA score – Creatinine (0-4)	2 [0 - 2]	
NMBA during first day, n (%)	20 (74.1)	
NMBA amount during first day, mg	175.5 (±217.8)	
NMBA average amount for the first 2 days, mg	107.3 (±136.1)	1
Cumulative fluid balance at day 1, L	5.6 (±3.7)	
ECMO, n (%)	3 (11.1)	0
VV-ECMO, n (%)	2 (7.4)	0
VA-ECMO, n (%)	1 (3.7)	0

**Table E2** - Patient demographics and critical illness data (Addition**a**l Data Table) (*continued*).		
Variable	***Result***	***N/a***
Number of reintubations (at least one), n (%)	5 (18.5)	0
1 reintubation	4 (14.8)	
2 reintubations	1 (3.7)	
**Blood tests**		
PaO_2/FiO_2, average of lowest values for each of the first 3 days, kPa	24 (±8.4)	3
Lactate, average of highest values for each of the first 3 days, mmol/L	3.9 (±3.7)	1
CRP, 24h highest value until first assessment, mg/L	223.8 (±144.1)	0
CRP, average of highest values for each of the first 3 days, mg/L	306.9 (±114.2)	13
Creatinine, 24h highest value until first assessment, µmol/L	190.3 (±106.9)	0
Creatinine, average of highest values for each of the first 3 days, µmol/L	172.6 (±104.2)	1
Bilirubin, 24h highest value until first assessment, µmol/L	40.8 (±55.6)	0
Bilirubin, average of highest values for each of the first 3 days, µmol/L	33 (±27.2)	6
Glycaemia, 24h highest value until first assessment, mmol/L	9.4 (±4)	0
Glycaemia, average of highest values for each of the first 3 days, mmol/L	9.6 (±2.3)	1
Glycaemia, 24h lowest value until first assessment, mmol/L	5.4 (±1.7)	0
Glycaemia, average of lowest values for each of the first 3 days, mmol/L	6.3 (±1)	1
Leucocyte count, 24h highest value until first assessment, g/L	13 (±8.1)	0

Values presented in percentage, mean (SD) or median [IQR] when applicable.

APACHE II, Acute Physiology and Chronic Health Evaluation II; BMI, Body Mass Index; CRP, C-Reactive Protein; CRRT, Continuous Renal Replacement Therapy; ECMO, Extracorporeal Membrane Oxygenation; FiO_2, Fraction of Inspired Oxygen; ICU, Intensive Care Unit; MAP, Mean Arterial Pressure; N/a, Missing Values; NMBA, Neuromuscular Blocking Agents; PaO_2, Arterial Oxygen Partial Pressure; PCT, Procalcitonin; SAPS, Simplified Acute Physiology Score; SAS, Sedation-Agitation Scale; SOFA, Sequential Organ Failure Assessment; VV-ECMO, Veno-Venous Extracorporeal Membrane Oxygenation; VA-ECMO, Veno-Arterial Extracorporeal Membrane Oxygenation.

**Table E3** – Data related to mobility and nutrition (Additional Data Table).		
Variable	***Result***	***N/a***
**Mobility**		
Moment for first active mobilisation session, n (%)		
Day 2	1 (3.7)	
Day 3	2 (7.4)	
Day 4	4 (14.8)	
Day 5	4 (14.8)	
After 5 days	16 (59.3)	
MRC sum score at first extubation, points	45 [34 - 50.8]	5

Values presented in percentage, mean (SD) or median [IQR] when applicable.

MRC, Medical Research Council; S5Q, Standardized Five Questions.

Table E4 – Reliability (ICC(A,3)) of *rectus femoris* muscle cross sectional area measurements at lower third region, by day of assessment.

Day	Region (side / landmark)	ICC [95% CI]	Reliability
1	Left / Lower third	0.995 [0.991, 0.998]	Excellent
1	Right / Lower third	0.997 [0.994, 0.999]	Excellent
2	Left / Lower third	0.996 [0.992, 0.998]	Excellent
2	Right / Lower third	0.995 [0.990, 0.998]	Excellent
3	Left / Lower third	0.993 [0.986, 0.997]	Excellent
3	Right / Lower third	0.996 [0.993, 0.998]	Excellent
4	Left / Lower third	0.995 [0.991, 0.998]	Excellent
4	Right / Lower third	0.995 [0.990, 0.997]	Excellent
5	Left / Lower third	0.992 [0.984, 0.997]	Excellent
5	Right / Lower third	0.995 [0.990, 0.998]	Excellent

CI, Confidence Interval ; ICC, Intraclass Correlation Coefficient.

Table E5 – Mean reliability of *rectus femoris* muscle cross sectional area measurements at lower third region.

Region (side / landmark)	Mean ICC	SD	Reliability
Left / Lower third	0.994	0.002	Excellent
Right / Lower third	0.996	0.001	Excellent

ICC – Intraclass Correlation Coefficient; SD, Standard Deviation.

**Table E6** – Reliability (ICC(A,3)) of *rectus femoris* muscle shear modulus measurements by day of assessment, stratified by ultrasound view and landmark.				
**Day**	**Type**	**Region (side / landmark)**	**ICC [95% CI]**	**Reliability**
1	Longitudinal	Left / Lower third	0.898 [0.799, 0.953]	Good
1	Transverse	Left / Lower third	0.930 [0.865, 0.966]	Excellent
1	Longitudinal	Right / Lower third	0.938 [0.879, 0.971]	Excellent
1	Transverse	Right / Lower third	0.866 [0.739, 0.936]	Good
1	Longitudinal	Left / Midpoint	0.888 [0.779, 0.949]	Good
1	Transverse	Left / Midpoint	0.771 [0.560, 0.890]	Good
1	Longitudinal	Right / Midpoint	0.991 [0.982, 0.996]	Excellent
1	Transverse	Right / Midpoint	0.909 [0.827, 0.956]	Excellent
2	Longitudinal	Left / Lower third	0.968 [0.933, 0.985]	Excellent
2	Transverse	Left / Lower third	0.950 [0.905, 0.976]	Excellent
2	Longitudinal	Right / Lower third	0.976 [0.952, 0.989]	Excellent
2	Transverse	Right / Lower third	0.900 [0.808, 0.952]	Good
2	Longitudinal	Left / Midpoint	0.941 [0.878, 0.974]	Excellent
2	Transverse	Left / Midpoint	0.950 [0.901, 0.977]	Excellent
2	Longitudinal	Right / Midpoint	0.972 [0.943, 0.988]	Excellent
2	Transverse	Right / Midpoint	0.950 [0.902, 0.977]	Excellent
3	Longitudinal	Left / Lower third	0.977 [0.954, 0.989]	Excellent
3	Transverse	Left / Lower third	0.887 [0.783, 0.946]	Good
3	Longitudinal	Right / Lower third	0.884 [0.767, 0.948]	Good
3	Transverse	Right / Lower third	0.911 [0.830, 0.957]	Excellent
3	Longitudinal	Left / Midpoint	0.900 [0.796, 0.956]	Excellent
3	Transverse	Left / Midpoint	0.829 [0.667, 0.919]	Good
3	Longitudinal	Right / Midpoint	0.941 [0.878, 0.974]	Excellent
3	Transverse	Right / Midpoint	0.908 [0.822, 0.956]	Excellent
4	Longitudinal	Left / Lower third	0.984 [0.966, 0.993]	Excellent
4	Transverse	Left / Lower third	0.956 [0.912, 0.980]	Excellent
4	Longitudinal	Right / Lower third	0.981 [0.962, 0.992]	Excellent
4	Transverse	Right / Lower third	0.967 [0.936, 0.985]	Excellent
4	Longitudinal	Left / Midpoint	0.938 [0.869, 0.973]	Excellent
4	Transverse	Left / Midpoint	0.906 [0.813, 0.957]	Excellent
4	Longitudinal	Right / Midpoint	0.953 [0.901, 0.980]	Excellent
4	Transverse	Right / Midpoint	0.947 [0.895, 0.976]	Excellent

**Table E6** – Reliability of *rectus femoris* muscle shear modulus measurements by day of assessment, stratified by ultrasound view and landmark. (*continued*)				
**Day**	**Type**	**Region (side / landmark)**	**ICC [95% CI]**	**Reliability**
5	Longitudinal	Left / Lower third	0.876 [0.730, 0.950]	Good
5	Transverse	Left / Lower third	0.956 [0.909, 0.981]	Excellent
5	Longitudinal	Right / Lower third	0.978 [0.952, 0.991]	Excellent
5	Transverse	Right / Lower third	0.939 [0.872, 0.974]	Excellent
5	Longitudinal	Left / Midpoint	0.955 [0.896, 0.983]	Excellent
5	Transverse	Left / Midpoint	0.888 [0.768, 0.951]	Good
5	Longitudinal	Right / Midpoint	0.979 [0.948, 0.993]	Excellent
5	Transverse	Right / Midpoint	0.965 [0.925, 0.985]	Excellent

CI, Confidence Interval ; ICC, Intraclass Correlation Coefficient.

**Table E7** – Mean reliability of *rectus femoris* muscle shear modulus measurements, stratified by ultrasound view and landmark.				
**Type**	**Region (side / landmark)**	**Mean ICC**	**SD**	**Reliability**
Longitudinal	Left / Lower third	0.940	0.050	Excellent
Transverse	Left / Lower third	0.936	0.029	Excellent
Longitudinal	Right / Lower third	0.952	0.042	Excellent
Transverse	Right / Lower third	0.916	0.039	Excellent
Longitudinal	Left / Midpoint	0.925	0.029	Excellent
Transverse	Left / Midpoint	0.869	0.069	Good
Longitudinal	Right / Midpoint	0.967	0.020	Excellent
Transverse	Right / Midpoint	0.936	0.026	Excellent

ICC – Intraclass Correlation Coefficient; SD, Standard Deviation.

**Table E8** – Percentage change in CSA_{RF}# from Day 1.					
**From day 1**	**Median [IQR]**	**n**	***p* value †**	**LMM estimated difference**	***p* value (LMM)**
Day 2	-3.75 [-12.9 to 5.5]	24	0.284	0.191	0.969
Day 3	-7.34 [-14.0 to 7.1]	24	0.141	-4.99	0.308
Day 4	**-15.00 [-24.8 to -6.00] ***	23	**0.014**	-9.04	0.072
Day 5	**-21.10 [-27.5 to -10.9] ****	20	**0.008**	**-12.80 ***	**0.015**

CSA_{RF} – Cross-Sectional Area of the *rectus femoris* muscle; IQR – Interquartile range, reported as Q1 to Q3; LMM – Linear mixed-effects model.

# CSA_{RF} at the lower third region. Values presented in percentage change from day 1.

† Comparison with Day 1 using the Wilcoxon signed-rank test.

LMM estimated difference represents the estimated mean difference in percentage change from Day 1, derived from linear mixed-effects models with day as a categorical fixed effect and patient as a random intercept. Day 1 was used as the reference category. LMM *p* value corresponds to the comparison between each day and Day 1 derived from the linear mixed-effects model. * *p* value < 0.05; ** *p* value < 0.01.

**Table E9** – Lower Third Region Summary Table								
**Metric**	**Day**	**N**	**n_ day**	**Mean (SD)**	**Median [IQR]**	***p* value †**	**LMM estimated difference**	***p* value (LMM)**
CSA_{RF} (cm^2)	1	N/a	25	3.92 (1.32)	3.90 [3.06 – 4.92]	*Ref.*	*Ref.*	*Ref.*
	2	24	25	3.86 (1.30)	4.03 [2.98 – 4.78]	0.271	-0.119	0.248
	3	24	25	3.71 (1.34)	3.74 [2.73 – 4.54]	0.077	**-0.267 ***	**0.011**
	4	23	25	3.49 (1.25)	**3.29 [2.84 – 4.54] ****	**0.002**	**-0.500 *****	**< 0.001**
	5	20	21	3.23 (1.22)	**2.97 [2.61 – 4.24] *****	**< 0.001**	**-0.648 *****	**< 0.001**
$ECHO_{RF}$ (greyscale units)	1	N/a	26	70.6 (18.5)	70.9 [57.6 – 81.8]	*Ref.*	*Ref.*	*Ref.*
	2	25	26	70.7 (14.3)	73.9 [58.9 – 79.9]	0.830	0.122	0.963
	3	25	26	69.9 (14.8)	70.9 [57.6 – 80.3]	0.830	-0.722	0.782
	4	24	25	69.1 (17.1)	70.5 [55.8 – 77.3]	0.520	-1.170	0.658
	5	21	22	70.1 (18.8)	66.1 [60.2 – 76.6]	0.651	-0.982	0.721
$SM_{RF-LONG}$ (kPa)	1	N/a	24	8.52 (4.28)	7.08 [5.92 – 9.44]	*Ref.*	*Ref.*	*Ref.*
	2	23	23	7.89 (4.11)	6.17 [5.33 – 8.26]	0.057	-0.720	0.238
	3	23	23	8.54 (5.25)	6.30 [5.12 – 10.70]	0.399	-0.061	0.920
	4	22	22	9.06 (5.05)	7.78 [5.57 – 9.53]	0.516	0.470	0.447
	5	18	18	9.52 (4.48)	7.72 [6.43 – 12.7]	0.925	0.457	0.488
$SM_{RF-TRANSV}$ (kPa)	1	N/a	26	15.50 (4.31)	15.20 [12.10 – 18.80]	*Ref.*	*Ref.*	*Ref.*
	2	25	26	16.70 (5.55)	15.70 [12.80 – 19.20]	0.510	1.240	0.216
	3	25	26	14.30 (4.89)	13.70 [10.60 – 17.30]	0.360	-1.160	0.249
	4	24	24	13.50 (6.84)	**11.90 [8.62 – 15.60] ***	**0.023**	**-2.190 ***	**0.034**
	5	20	21	12.60 (6.43)	**12.30 [8.33 – 14.40] ****	**0.009**	**-3.35 ****	**0.002**
$SM_{RF-TRANSV/LONG\ RATIO}$ (ratio)	1	N/a	24	2.16 (0.97)	2.12 [1.32 – 2.72]	*Ref.*	*Ref.*	*Ref.*
	2	23	23	2.55 (1.21)	**2.38 [1.82 – 3.09] ***	**0.012**	1.167	0.073
	3	23	23	2.10 (0.82)	2.14 [1.56 – 2.81]	0.616	0.997	0.970
	4	22	22	1.71 (0.72)	**1.70 [1.34 – 2.21] ***	**0.010**	**0.784 ****	**0.006**
	5	18	18	1.56 (0.66)	**1.50 [1.05 – 2.15] ***	**0.019**	**0.718 *****	**< 0.001**

CSA_{RF} – Cross-sectional area of the *rectus femoris* muscle (in cm²); $ECHO_{RF}$ – Echogenicity of the *rectus femoris* muscle (greyscale units, 0 to 255); IQR – Interquartile range, reported as Q1 to Q3; LMM – Linear mixed-effects model; *Ref.* – Reference value; SD – Standard deviation; $SM_{RF-LONG}$ – Shear modulus of the *rectus femoris* muscle measured in the longitudinal ultrasound view (in kPa); $SM_{RF-TRANSV}$ – Shear modulus of the *rectus femoris* muscle measured in the transverse ultrasound view (in kPa); $SM_{RF-TRANSV/LONG\ RATIO}$ – Ratio of Shear modulus values of the *rectus femoris* muscle acquired in the transverse plane relative to the longitudinal plane.

N refers to the sample size included in the statistical test for that day, and *p* value corresponds to the *p*-value associated with the test comparing that day to Day 1. n_day denotes the number of available data points for the respective metric on that specific day. LMM estimated difference: mean difference from Day 1, expressed in the original unit of each variable, derived from linear mixed-effects models with day as categorical fixed effect (Day 1

**Table E9** – Lower Third Region Summary Table								
**Metric**	**Day**	**N**	**n_ day**	**Mean (SD)**	**Median [IQR]**	***p* value †**	**LMM estimated difference**	***p* value (LMM)**

as the reference category) and participant as a random intercept. LMM p value: the corresponding comparison between each day and Day 1. For the transverse-to-longitudinal shear-modulus ratio, the model was fitted on log-transformed values and the coefficient is presented back-transformed; it is therefore a ratio (Day k relative to Day 1) rather than a difference, with 1 indicating no change from Day 1.

† Comparison with Day 1 using the Wilcoxon signed-rank test.

* *p* value < 0.05; ** *p* value < 0.01; *** *p* value < 0.001.

**Table E10** – Midpoint Region Summary Table								
**Metric**	**Day**	**N**	**n_ day**	**Mean (SD)**	**Median [IQR]**	***p* value †**	**LMM estimated difference**	***p* value (LMM)**
$ECHO_{RF}$ (greyscale units)	1	N/a	25	53.3 (16.2)	51.4 [41.1 – 64.0]	*Ref.*	*Ref.*	*Ref.*
	2	24	25	52.7 (16.4)	51.9 [40.5 – 62.7]	1.000	-0.176	0.935
	3	24	25	52.7 (16.4)	47.8 [38.8 – 65.9]	0.520	-0.973	0.651
	4	24	25	56.1 (17.5)	52.8 [40.9 – 69.8]	0.658	3.160	0.145
	5	21	21	53.0 (17.3)	52.5 [40.2 – 62.7]	0.808	-0.048	0.983
$SM_{RF-LONG}$ (kPa)	1	N/a	23	12.40 (9.72)	8.37 [7.23 – 11.7]	*Ref.*	*Ref.*	*Ref.*
	2	21	21	13.30 (11.70)	8.70 [6.78 – 11.70]	0.835	1.110	0.620
	3	21	21	14.80 (13.70)	9.10 [6.85 – 17.60]	0.313	2.610	0.245
	4	21	21	14.60 (15.40)	8.92 [6.38 – 12.80]	0.651	1.920	0.393
	5	17	17	17.80 (19.20)	9.30 [6.78 – 16.10]	0.887	3.81	0.115
$SM_{RF-TRANSV}$ (kPa)	1	N/a	26	13.10 (5.36)	11.20 [9.53 – 15.20]	*Ref.*	*Ref.*	*Ref.*
	2	24	24	16.80 (12.60)	**12.30 [9.47 – 19.10] ***	**0.041**	**3.960 ***	**0.012**
	3	24	25	16.20 (12.90)	11.30 [8.92 – 19.20]	0.141	**3.570 ***	**0.021**
	4	24	24	15.80 (13.10)	10.90 [7.24 – 16.70]	0.764	2.51	0.105
	5	20	21	14.10 (10.90)	10.70 [6.92 – 15.70]	0.926	0.459	0.776
$SM_{RF-TRANSV/LONG\ RATIO}$ (ratio)	1	N/a	23	1.36 (0.61)	1.45 [0.78 – 1.72]	*Ref.*	*Ref.*	*Ref.*
	2	21	21	1.58 (0.72)	1.31 [1.10 – 1.78]	0.102	1.175	0.134
	3	21	21	1.43 (0.67)	1.16 [0.99 – 1.96]	0.444	1.05	0.642
	4	21	21	1.42 (0.67)	1.32 [0.93 – 1.60]	0.889	1.023	0.832
	5	17	17	1.12 (0.57)	0.97 [0.70 – 1.33]	0.508	0.826	0.097

CSA_{RF} – Cross-Sectional Area of the *rectus femoris* muscle (in cm²); $ECHO_{RF}$ – Echogenicity of the *rectus femoris* muscle (greyscale, 0 to 255); IQR – Interquartile range, reported as Q1 to Q3; LMM – Linear mixed-effects model; *Ref.* – Reference value; SD – Standard deviation; $SM_{RF-LONG}$ – Shear Modulus of the *rectus femoris* muscle measured in the longitudinal ultrasound view (in kPa); $SM_{RF-TRANSV}$ – Shear Modulus of the *rectus femoris* muscle measured in the transverse ultrasound view (in kPa); $SM_{RF-TRANSV/LONG\ RATIO}$ – Ratio of Shear Modulus values of the *rectus femoris* muscle acquired in the transverse plane relative to the longitudinal plane.

N refers to the sample size included in the statistical test for that day, and *p* value corresponds to the p-value associated with the test comparing that day to Day 1. n_day denotes the number of available data points for the respective metric on that specific day. LMM estimated difference: mean difference from Day 1, expressed in the original unit of each variable, derived from linear mixed-effects models with day as a categorical fixed effect (Day 1 as the reference category) and participant as a random intercept. LMM *p* value: the corresponding comparison between each day and Day 1. For the transverse-to-longitudinal shear-modulus ratio, the model was fitted on log-transformed values, and the coefficient is presented back-transformed; it is therefore a ratio (Day k relative to Day 1) rather than a difference, with 1 indicating no change from Day 1.

† Comparison with Day 1 using the Wilcoxon signed-rank test. * *p* value < 0.05.

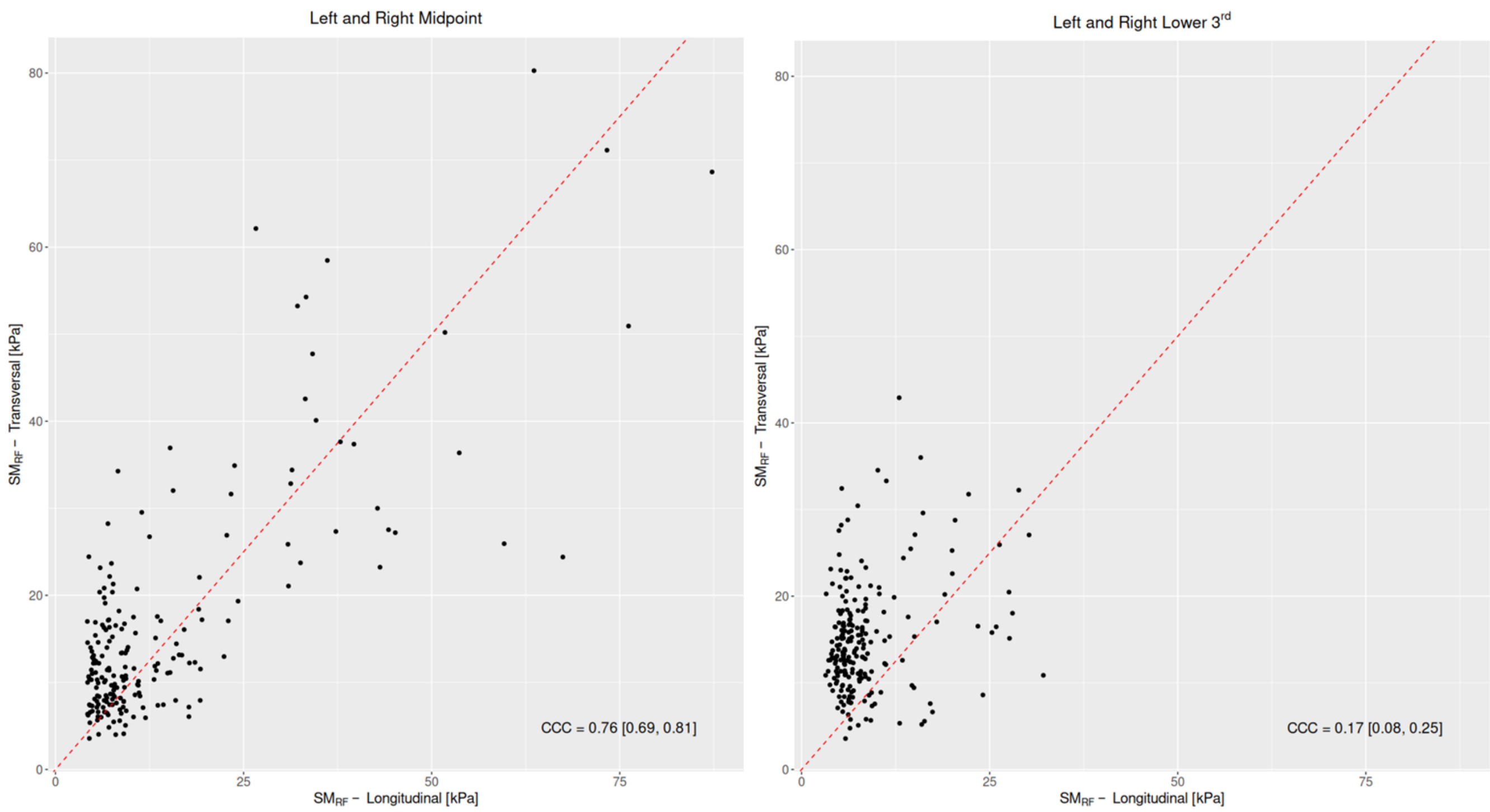

Figure E2 – CCC (95% confidence interval) between SM_{RF} Transverse and Longitudinal views for mid- (left graph) and lower third (right graph) anatomical points of assessment.

CCC, Concordance Correlation Coefficient; SM_{RF}, Shear modulus of *rectus femoris* muscle.

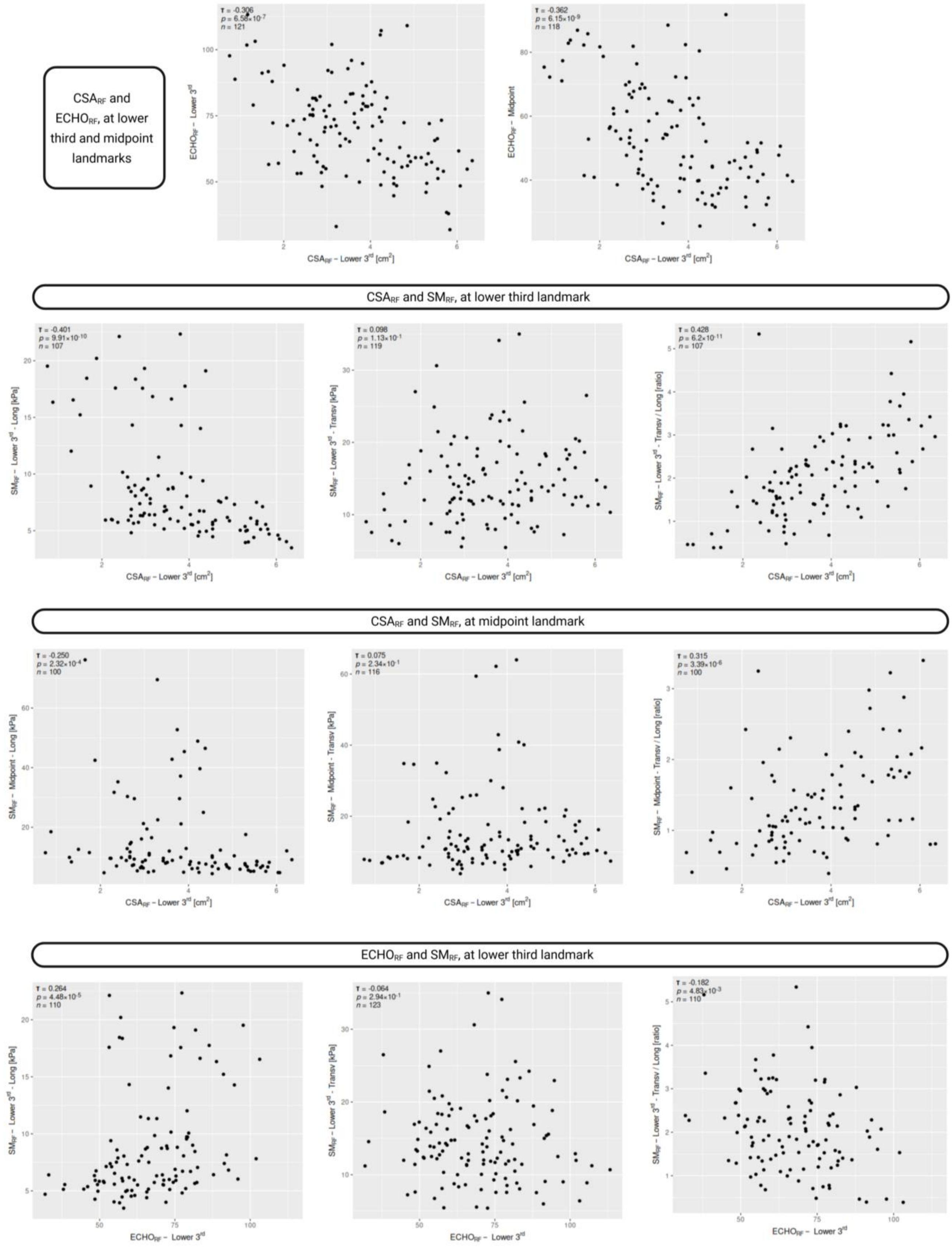

Figure E3 – Overall correlations between CSA_{RF}, $ECHO_{RF}$, SM_{RF} (in both longitudinal and transverse views), at both lower third and midpoint landmarks.

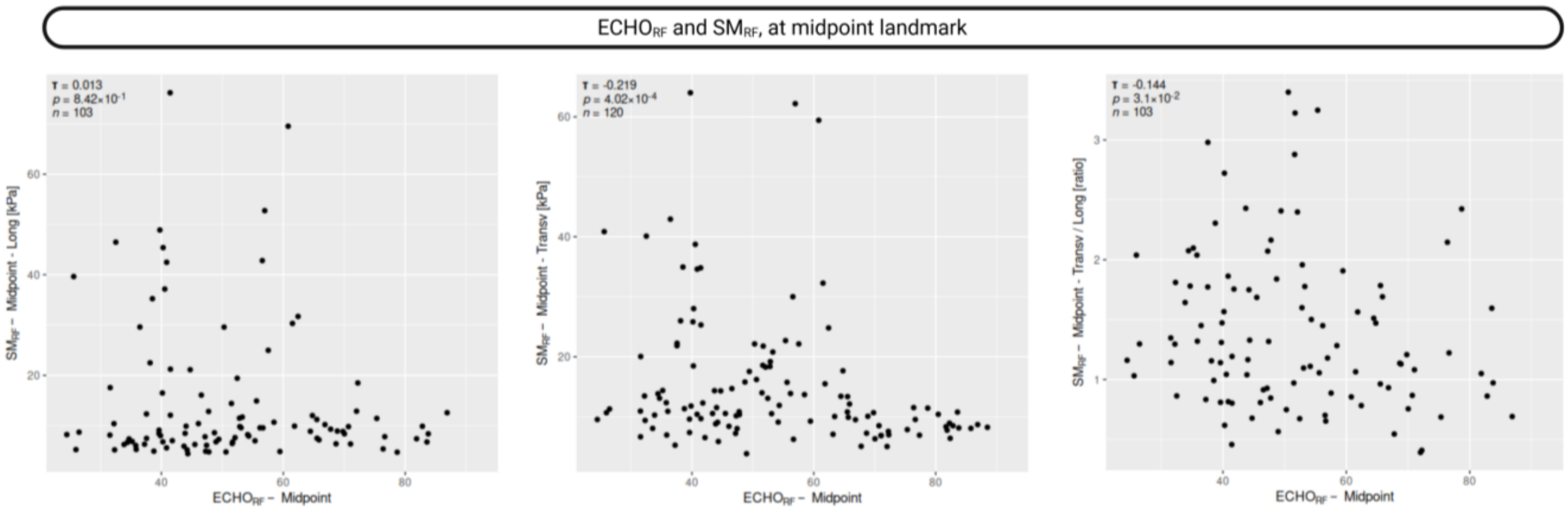

Figure E3 (*continued*)– Overall correlations between CSA_{RF}, $ECHO_{RF}$, SM_{RF} (in both longitudinal and transverse views), at both lower third and midpoint landmarks.

CSA_{RF} – Cross-Sectional Area of the *rectus femoris* muscle (in cm²); $ECHO_{RF}$ – Echogenicity of the *rectus femoris* muscle (greyscale, 0 to 255); SM_{RF} – Shear Modulus of the *rectus femoris* muscle (in kPa); TRANSV/LONG – ratio between transverse and longitudinal view assessment of shear modulus. τ – Kendall's tau correlation coefficient; *p* – p-value; *n* – number of paired observations.

**Table E11** – Correlation by Day and Region.					
**Landmark**	**Day**	Lower third		Midpoint	
		**Kendall's tau**	***p* value**	**Kendall's tau**	***p* value**
CSA_{RF}# vs $SM_{RF\text{-}LONG}$	1	**−0.368 ***	**0.014**	−0.217	0.158
	2	**−0.429 ****	**0.005**	−0.295	0.074
	3	**−0.558 *****	**< 0.001**	−0.253	0.128
	4	**−0.420 ****	**0.006**	−0.257	0.110
	5	−0.333	0.057	−0.353	0.052
CSA_{RF}# vs $SM_{RF\text{-}TRANSV}$	1	0.057	0.691	0.043	0.761
	2	0.113	0.445	0.000	1.000
	3	0.077	0.591	0.065	0.677
	4	−0.015	0.941	0.116	0.446
	5	0.126	0.461	0.116	0.501
$ECHO_{RF}$ vs $SM_{RF\text{-}LONG}$	1	0.239	0.107	0.119	0.428
	2	**0.296 ***	**0.0498**	0	1.000
	3	0.289	0.0566	−0.0952	0.571
	4	**0.359 ***	**0.0194**	−0.0286	0.882
	5	0.307	0.0813	0.191	0.308
$ECHO_{RF}$ vs $SM_{RF\text{-}TRANSV}$	1	−0.031	0.826	−0.0772	0.581
	2	−0.046	0.760	−0.142	0.333
	3	−0.222	0.112	**−0.420 ****	**0.002**
	4	0.029	0.864	−0.268	0.0698
	5	−0.229	0.158	−0.124	0.455

CSA_{RF} – Cross-Sectional Area of the *rectus Femoris* muscle (in cm²); $ECHO_{RF}$ – Echogenicity of the *rectus femoris* muscle (greyscale units, 0 to 255); $SM_{RF\text{-}LONG}$ – Shear Modulus of the *rectus Femoris* muscle measured in the longitudinal ultrasound view (in kPa); $SM_{RF\text{-}TRANSV}$ – Shear Modulus of the *rectus Femoris* muscle measured in the transverse ultrasound view (in kPa).

#It concerns the assessment of CSA_{RF} at the lower third region.

* *p* value < 0.05; ** *p* value < 0.01; *** *p* value < 0.001.

**Table E12** – Percentage change in CSA_{RF}[#] from Day 1, categorised by weakness status									
**From day 1**	**Weakness (MRC-SS <48)**				**No weakness (MRC-SS ≥48)**				***p* value ‡**
	**Mean (SD)**	**Median [IQR]**	n	***p* value †**	**Mean (SD)**	**Median [IQR]**	n	***p* value †**	
Day 2	1.46 (±18.00)	-0.93 [-6.76 to 4.26]	11	0.756	-7.47 (±12.70)	-9.16 [-17.50 to -3.07]	10	0.126	0.197
Day 3	-5.62 (±14.20)	-8.34 [-13.00 to -5.15]	11	0.142	-6.84 (±17.40)	-4.32 [-22.50 to 5.37]	10	0.308	0.918
Day 4	-9.03 (±18.20)	-12.60 [-21.20 to -6.44]	11	0.068	-15.40 (±16.90)	**-21.00 [-25.40 to -5.66] ***	9	**0.033**	0.503
Day 5	-20.3 (±12.60)	**-21.00 [-26.70 to -15.90] ****	9	**0.009**	-20.60 (±14.30)	**-23.40 [-26.70 to -17.20] ***	8	**0.021**	0.888

CSA_{RF} – Cross-sectional area of the *rectus femoris* muscle; IQR – Interquartile range, reported as Q1 to Q3; MRC – Medical Research Council sum score; SD – Standard deviation.

[#] CSA_{RF} at the lower third region. Values presented in percentage change from day 1.

† Comparison with Day 1 using the Wilcoxon signed-rank test.

‡ Wilcoxon rank-sum test used for comparison between weakness groups at each time point.

* *p* value < 0.05; ** *p* value < 0.01.

Table E13 – CSA_{RF}, $ECHO_{RF}$ and SM_{RF} (absolute values) over the first 5 days at lower third landmark, categorised by weakness status.

Metrics	Day	Subgroup	n	Mean (SD)	Median [IQR]	Comparison from day 1, by group †	LMM estimated difference	*p* value (LMM)	Between groups comparison, per day ‡
CSA_{RF} (in cm^2)	1	MRC-SS <48	11	4.37 (±1.19)	4.34 [3.66 – 5.30]	*Ref.*	*Ref.*	*Ref.*	0.512
		MRC-SS ≥48	10	4.15 (±0.96)	4.06 [3.60 – 4.77]	*Ref.*	*Ref.*	*Ref.*	
	2	MRC-SS <48	11	4.37 (±1.26)	4.21 [3.27 – 5.60]	0.898	0.005	0.974	0.223
		MRC-SS ≥48	10	3.81 (±0.93)	3.92 [3.43 – 4.07]	0.065	**-0.337 ***	**0.031**	
	3	MRC-SS <48	11	4.10 (±1.23)	3.93 [3.01 – 4.92]	0.175	-0.270	0.068	0.705
		MRC-SS ≥48	10	3.84 (±1.16)	3.74 [2.93 – 4.30]	0.432	**-0.309 ***	**0.047**	
	4	MRC-SS <48	12	3.82 (±1.12)	**3.50 [2.89 – 4.58] ***	**0.032**	**-0.477 ****	**0.002**	0.917
		MRC-SS ≥48	9	3.58 (±1.00)	**3.63 [3.02 – 3.95] ***	**0.019**	**-0.687 *****	**< 0.001**	
	5	MRC-SS <48	10	3.62 (±1.26)	**3.24 [2.65 – 4.46] ****	**0.004**	**- 0.769 *****	**< 0.001**	0.965
		MRC-SS ≥48	8	3.30 (±0.92)	**3.16 [2.96 – 3.78] ***	**0.016**	**-0.760 *****	**< 0.001**	
$ECHO_{RF}$ (greyscale units)	1	MRC-SS <48	12	67.8 (±21.5)	68.7 [55.8 – 79.0]	*Ref.*	*Ref.*	*Ref.*	0.771
		MRC-SS ≥48	10	66.0 (±11.6)	62.9 [57.6 – 71.7]	*Ref.*	*Ref.*	*Ref.*	
	2	MRC-SS <48	12	69.7 (±15.5)	74.6 [57.7 – 78.4]	0.970	1.830	0.607	0.974
		MRC-SS ≥48	10	69.5 (±15.2)	64.4 [58.0 – 81.0]	0.275	3.47	0.376	
	3	MRC-SS <48	12	64.8 (±10.8)	62.1 [57.3 – 75.1]	0.622	-3.050	0.393	0.539
		MRC-SS ≥48	10	67.9 (±13.8)	70.9 [55.4 – 80.3]	0.432	1.910	0.625	
	4	MRC-SS <48	12	64.9 (±12.1)	67.0 [55.7 – 74.0]	0.569	-2.970	0.406	0.862
		MRC-SS ≥48	9	64.0 (±15.2)	58.1 [53.5 – 72.5]	0.910	-1.040	0.798	
	5	MRC-SS <48	10	66.6 (±12.3)	65.7 [62.1 – 74.3]	0.557	-0.986	0.794	0.897
		MRC-SS ≥48	8	65.9 (±19.8)	66.0 [56.2 – 73.6]	0.742	-0.424	0.920	
$SM_{RF-LONG}$ (in kPa)	1	MRC-SS <48	11	7.40 (±2.11)	6.82 [5.76 – 8.72]	*Ref.*	*Ref.*	*Ref.*	0.705
		MRC-SS ≥48	10	9.15 (±5.02)	7.89 [6.16 – 9.23]	*Ref.*	*Ref.*	*Ref.*	
	2	MRC-SS <48	11	7.01 (±4.25)	5.75 [5.03 – 7.05]	0.175	-0.394	0.634	0.099
		MRC-SS ≥48	10	8.65 (±4.07)	6.78 [6.12 – 9.40]	0.375	-0.497	0.567	

Table E13 – CSA_{RF}, $ECHO_{RF}$ and SM_{RF} (absolute values) over the first 5 days at lower third landmark, categorised by weakness status.

Metrics	Day	Subgroup	n	Mean (SD)	Median [IQR]	Comparison from day 1, by group †	LMM estimated difference	*p* value (LMM)	Between groups comparison, per day ‡
	3	MRC-SS <48	11	6.38 (±2.68)	**5.73 [4.75 – 6.59] ***	**0.032**	-1.030	0.216	0.251
		MRC-SS ≥48	10	9.99 (±6.65)	6.37 [5.29 – 14.70]	0.846	0.842	0.333	
	4	MRC-SS <48	11	7.59 (±3.65)	5.92 [5.09 – 9.16]	0.831	0.188	0.820	0.295
		MRC-SS ≥48	9	10.20 (±6.42)	8.05 [5.92 – 8.72]	0.250	0.980	0.820	
	5	MRC-SS <48	8	8.11 (±4.09)	7.12 [5.85 – 8.25]	1.000	0.420	0.648	0.382
		MRC-SS ≥48	8	9.50 (±4.41)	7.72 [6.50 – 10.10]	1.000	-0.149	0.873	
$SM_{RF-TRANSV}$ (in kPa)	1	MRC-SS <48	12	15.70 (±4.01)	14.80 [12.00 – 19.10]	*Ref.*	*Ref.*	*Ref.*	0.974
		MRC-SS ≥48	10	15.80 (±4.97)	15.30 [12.70 – 19.50]	*Ref.*	*Ref.*	*Ref.*	
	2	MRC-SS <48	12	15.60 (±4.49)	14.80 [13.50 – 16.80]	0.814	-0.101	0.945	0.381
		MRC-SS ≥48	10	17.60 (±5.36)	18.20 [13.10 – 22.10]	0.275	1.770	0.269	
	3	MRC-SS <48	12	13.10 (±4.12)	12.20 [10.10 – 17.30]	0.092	-2.630	0.074	0.070
		MRC-SS ≥48	10	17.00 (±4.76)	14.80 [14.20 – 20.30]	0.275	1.200	0.453	
	4	MRC-SS <48	12	12.0 (±3.44)	**11.60 [9.81 – 13.20] ****	**0.007**	**-3.670 ***	**0.014**	0.651
		MRC-SS ≥48	9	16.2 (±9.96)	13.90 [7.62 – 23.80]	1.000	-3.670	0.982	
	5	MRC-SS <48	9	12.60 (±2.97)	**13.10 [11.40 – 13.40] ***	**0.039**	**-3.620 ***	**0.026**	0.606
		MRC-SS ≥48	8	13.60 (±9.29)	12.10 [7.93 – 14.40]	0.195	-3.080	0.077	
$SM_{RF-TRANSV/LONG\ RATIO}$ (ratio)	1	MRC-SS <48	11	2.33 (±0.82)	2.28 [1.97 – 2.80]	*Ref.*	*Ref.*	*Ref.*	0.314
		MRC-SS ≥48	10	2.09 (±1.12)	1.72 [1.20 – 2.71]	*Ref.*	*Ref.*	*Ref.*	
	2	MRC-SS <48	11	2.79 (±1.23)	**2.89 [2.08 – 3.31] ***	**0.042**	1.110	0.398	0.152
		MRC-SS ≥48	10	2.20 (±0.66)	2.31 [1.66 – 2.80]	0.375	1.140	0.342	
	3	MRC-SS <48	11	2.28 (±0.82)	2.14 [1.69 – 2.96]	0.898	0.981	0.881	0.756
		MRC-SS ≥48	10	2.10 (±0.75)	2.21 [1.45 – 2.60]	1.000	1.06	0.672	
	4	MRC-SS <48	11	1.89 (±0.77)	**1.84 [1.48 – 2.31] ***	**0.032**	0.785	0.059	0.370
		MRC-SS ≥48	9	1.64 (±0.59)	1.53 [1.34 – 1.89]	0.250	0.800	0.108	

**Table E13** – CSA_{RF}, $ECHO_{RF}$ and SM_{RF} (absolute values) over the first 5 days at lower third landmark, categorised by weakness status.									
**Metrics**	**Day**	**Subgroup**	**n**	**Mean (SD)**	**Median [IQR]**	**Comparison from day 1, by group** †	**LMM estimated difference**	***p* value (LMM)**	**Between groups comparison, per day** ‡
	5	MRC-SS <48	8	1.85 (±0.59)	1.95 [1.51 – 2.25]	0.109	0.768	0.063	0.279
		MRC-SS ≥48	8	1.43 (±0.64)	1.24 [0.99 – 1.72]	0.195	**0.696 ***	**0.013**	

CSA_{RF} – Cross-Sectional Area of the *rectus femoris* muscle (in cm²); $ECHO_{RF}$ – Echogenicity of the *rectus femoris* muscle (greyscale units, 0 to 255); IQR – Interquartile range, reported as Q1 to Q3; LMM – linear mixed-effects model; MRC – Medical Research Council sum score; *Ref.* – Reference value; SD – Standard deviation; $SM_{RF\text{-}LONG}$ – Shear Modulus of the *Rectus Femoris* muscle measured in the longitudinal ultrasound view (in kPa); $SM_{RF\text{-}TRANSV}$ – Shear Modulus of the *rectus femoris* muscle measured in the transverse ultrasound view (in kPa); $SM_{RF\text{-}TRANSV/LONG\ RATIO}$ – Ratio of Shear Modulus values of the *rectus femoris* muscle acquired in the transverse plane relative to the longitudinal plane.

LMM estimated difference: mean difference from Day 1 within each MRC subgroup, expressed in the original unit of each variable, derived from a single linear mixed-effects model containing day (categorical, Day 1 as the reference category), MRC group and their interaction, with participant as a random intercept; within-subgroup changes were obtained as estimated marginal means. LMM p value: the corresponding comparison between each day and Day 1 within that subgroup. For the transverse-to-longitudinal shear-modulus ratio, the model was fitted on log-transformed values and the coefficient is presented back-transformed; it is therefore a ratio (Day k relative to Day 1) rather than a difference, with 1 indicating no change from Day 1.

† Comparison with Day 1 using the Wilcoxon signed-rank test.

‡ Wilcoxon rank-sum test used for comparison between weakness groups at each time point.

* *p* value < 0.05; ** *p* value < 0.01; *** *p* value < 0.001.

Table E14 – ECHO$_{RF}$ and SM$_{RF}$ (absolute values) over the first 5 days at midpoint landmark, categorised by weakness status.									
**Metric**	**Day**	**Subgroup**	**n**	**Mean (SD)**	**Median [IQR]**	**Comparison from day 1, by group †**	**LMM estimated difference**	***p* value (LMM)**	**Between groups comparison, per day ‡**
ECHO$_{RF}$ (greyscale units)	1	MRC-SS <48	12	52.8 (±18.1)	50.6 [42.9 – 62.3]	*Ref.*	*Ref.*	*Ref.*	0.456
		MRC-SS ≥48	10	47.5 (±11.6)	43.6 [40.4 – 53.2]	*Ref.*	*Ref.*	*Ref.*	
	2	MRC-SS <48	12	51.0 (±13.5)	51.9 [41.0 – 54.6]	0.622	-1.810	0.568	0.702
		MRC-SS ≥48	9	47.7 (±15.4)	44.2 [40.2 – 62.4]	1.000	0.944	0.793	
	3	MRC-SS <48	12	53.2 (±15.7)	51.6 [39.2 – 66.1]	0.910	0.334	0.916	0.193
		MRC-SS ≥48	9	45.1 (±15.0)	43.4 [38.5 – 47.3]	0.359	-1.670	0.642	
	4	MRC-SS <48	12	54.9 (±15.0)	55.0 [42.8 – 68.9]	0.791	2.120	0.504	0.310
		MRC-SS ≥48	9	48.8 (±15.5)	42.1 [36.5 – 64.4]	0.426	4.050	0.261	
	5	MRC-SS <48	10	54.1 (±17.1)	53.0 [44.9 – 61.3]	0.232	1.790	0.593	0.122
		MRC-SS ≥48	8	43.1 (±13.1)	39.3 [35.2 – 50.9]	0.461	-1.700	0.649	
SM$_{RF-LONG}$ (in kPa)	1	MRC-SS <48	11	11.40 (±6.97)	8.22 [7.63 – 14.9]	*Ref.*	*Ref.*	*Ref.*	0.939
		MRC-SS ≥48	9	14.10 (±13.90)	8.20 [6.25 – 9.90]	*Ref.*	*Ref.*	*Ref.*	
	2	MRC-SS <48	11	13.60 (±12.40)	9.58 [7.18 – 11.90]	0.577	2.200	0.481	0.600
		MRC-SS ≥48	8	14.10 (±12.70)	8.09 [6.61 – 15.30]	0.742	0.205	0.954	
	3	MRC-SS <48	11	13.90 (±13.70)	9.10 [7.01 – 12.30]	0.577	2.500	0.424	0.717
		MRC-SS ≥48	8	16.20 (±16.00)	7.92 [4.91 – 22.00]	0.945	2.310	0.424	
	4	MRC-SS <48	11	14.30 (±18.70)	8.83 [5.63 – 12.60]	0.700	2.890	0.355	0.492
		MRC-SS ≥48	8	15.50 (±13.30)	8.89 [8.03 – 17.00]	0.547	0.712	0.843	
	5	MRC-SS <48	8	11.30 (±8.47)	8.65 [6.77 – 11.20]	0.313	-1.900	0.584	0.397
		MRC-SS ≥48	7	27.00 (±26.90)	11.20 [6.54 – 41.20]	0.469	**11.000 ***	**0.005**	
SM$_{RF-TRANSV}$ (in kPa)	1	MRC-SS <48	12	13.20 (±4.74)	11.70 [9.53 – 14.70]	*Ref.*	*Ref.*	*Ref.*	0.895
		MRC-SS ≥48	10	13.50 (±6.64)	10.70 [10.30 – 14.70]	*Ref.*	*Ref.*	*Ref.*	
	2	MRC-SS <48	12	18.80 (±15.10)	13.80 [10.40 – 19.10]	0.064	**5.580 ***	**0.019**	0.310
		MRC-SS ≥48	9	15.40 (±10.50)	11.30 [8.50 – 18.50]	0.652	2.200	0.405	
	3	MRC-SS <48	12	17.10 (±15.40)	12.60 [8.97 – 16.20]	0.470	3.920	0.095	0.972

Table E14 – $ECHO_{RF}$ and SM_{RF} (absolute values) over the first 5 days at midpoint landmark, categorised by weakness status.

Metric	Day	Subgroup	n	Mean (SD)	Median [IQR]	Comparison from day 1, by group †	LMM estimated difference	*p* value (LMM)	Between groups comparison, per day ‡
		MRC-SS ≥48	9	17.10 (±12.20)	10.50 [10.10 – 20.00]	0.359	3.880	0.144	
	4	MRC-SS <48	12	17.30 (±14.40)	12.50 [10.30 – 17.60]	0.850	4.120	0.079	0.247
		MRC-SS ≥48	9	15.30 (±13.70)	10.10 [6.95 – 13.40]	0.820	0.936	0.723	
	5	MRC-SS <48	9	12.50 (±8.53)	10.70 [6.23 – 14.70]	0.820	-1.790	0.485	0.673
		MRC-SS ≥48	8	18.20 (±14.60)	11.60 [7.35 – 31.20]	0.641	3.130	0.255	
$SM_{RF-TRANSV/LONG\ RATIO}$ (ratio)	1	MRC-SS <48	11	1.45 (±0.75)	1.22 [0.85 – 1.82]	*Ref.*	*Ref.*	*Ref.*	0.766
		MRC-SS ≥48	9	1.30 (±0.50)	1.56 [0.76 – 1.69]	*Ref.*	*Ref.*	*Ref.*	
	2	MRC-SS <48	11	1.60 (±0.63)	1.31 [1.17 – 1.93]	0.700	1.160	0.330	0.657
		MRC-SS ≥48	8	1.45 (±0.63)	1.31 [1.00 – 1.76]	0.250	1.130	0.486	
	3	MRC-SS <48	11	1.46 (±0.71)	1.17 [1.03 – 1.74]	0.966	1.040	0.793	0.968
		MRC-SS ≥48	8	1.45 (±0.61)	1.10 [1.03 – 2.09]	0.250	1.140	0.455	
	4	MRC-SS <48	11	1.69 (±0.77)	1.57 [1.17 – 2.03]	0.465	1.20	0.226	0.109
		MRC-SS ≥48	8	1.11 (±0.38)	1.22 [0.90 – 1.37]	0.250	0.828	0.267	
	5	MRC-SS <48	8	1.30 (±0.65)	1.20 [0.85 – 1.61]	0.461	0.925	0.634	0.281
		MRC-SS ≥48	7	0.94 (±0.53)	0.83 [0.63 – 1.00]	0.109	**0.691***	**0.039**	

$SM_{RF-LONG}$ – Shear Modulus of the *Rectus Femoris* muscle measured in the longitudinal ultrasound view (in kPa); $ECHO_{RF}$ – Echogenicity of the *rectus femoris* muscle (greyscale units, 0 to 255); IQR – Interquartile range, reported as Q1 to Q3; LMM – linear mixed-effects model; MRC – Medical Research Council sum score; *Ref.* – Reference value; SD – Standard deviation; $SM_{RF-TRANSV}$ – Shear Modulus of the *rectus femoris* muscle measured in the transverse ultrasound view (in kPa); $SM_{RF-TRANSV/LONG\ RATIO}$ – Ratio of Shear Modulus values of the *rectus femoris* muscle acquired in the transverse plane relative to the longitudinal plane.

LMM estimate vs Day 1: mean difference from Day 1 within each MRC subgroup, expressed in the original unit of each variable, derived from a single linear mixed-effects model containing day (categorical, Day 1 as the reference category), MRC group and their interaction, with participant as a random intercept; within-subgroup changes were obtained as estimated marginal means. LMM p value: the corresponding comparison between each day and Day 1 within that subgroup. For the transverse-to-longitudinal shear-modulus ratio, the model was fitted on log-transformed values and the coefficient is presented back-transformed; it is therefore a ratio (Day k relative to Day 1) rather than a difference, with 1 indicating no change from Day 1.

† Comparison with Day 1 using the Wilcoxon signed-rank test.

**Table E14** – ECHO$_{RF}$ and SM$_{RF}$ (absolute values) over the first 5 days at midpoint landmark, categorised by weakness status.									
**Metric**	**Day**	**Subgroup**	**n**	**Mean (SD)**	**Median [IQR]**	**Comparison from day 1, by group** †	**LMM estimated difference**	***p* value (LMM)**	**Between groups comparison, per day** ‡

‡ Wilcoxon rank-sum test used for comparison between weakness groups at each time point.

* *p* value < 0.05.

**Table E15** – Univariate linear regressions between percentage changes in ultrasound-derived *rectus femoris* parameters and selected clinical variables.					
**Variable**	**β (SE)**	**95% CI**	***p*-value**	**R^2**	**N**
**% change CSA_{RF} from day 1 to day 5, at lower third point**					
SOFA score [points], at admission – linear model (after exclusion)	1.881 (2.700)	[−3.816, 7.578]	0.495	0.028	19
Mean protein intake [g/day] (first 3 days) – linear model (after exclusion, BCa bootstrap applied)	−0.763 (0.421) §	[−1.661, 0.036]	0.096	0.173	19
Highest daily glycaemia level [mmol/l] (average over the first 3 days) – linear model (after exclusion)	−0.936 (2.015)	[−5.220, 3.350]	0.648	0.013	19
**% change $ECHO_{RF}$ from day 1 to day 5, at midpoint**					
Mean caloric intake [kcal] (first 3 days) – linear model (after exclusion)	0.030 (0.013) *	[0.005, 0.055]	0.032	0.230	20
Mean lactate [mmol/L] – linear model (after exclusion)	0.546 (2.195)	[−4.108, 5.199]	0.807	0.004	18
**% change $SM_{RF-LONG}$ from day 1 to day 3, at midpoint**					
SOFA score [points], at admission – linear model (after exclusion, BCa bootstrap applied)	18.178 (4.307) **	[10.010, 27.161]	0.003	0.436	19
**% change $SM_{RF-LONG}$ from day 1 to day 3, at lower third point**					
SOFA score [points], at admission – linear model (after exclusion, BCa bootstrap applied)	8.453 (4.544) §	[−0.887, 14.601]	0.083	0.215	21
Cumulative fluid balance at Day 3 [Litres] – linear model (after exclusion, BCa bootstrap applied)	3.316 (0.992) **	[1.124, 4.306]	0.002	0.414	21
**% change $SM_{RF-TRANSV}$ from day 1 to day 3, at midpoint**					
Mean Caloric intake [kcal] (first 3 days) – linear model (after exclusion, BCa bootstrap applied)	0.076 (0.026) **	[0.029, 0.126]	0.006	0.252	23
Mean CRP [mg/l] (first 3 days) – linear model (after exclusion, BCa bootstrap applied)	0.181 (0.068) *	[0.050, 0.335]	0.016	0.217	22
Δ% $ECHO_{RF}$ [greyscale units] (lower third, Day 1–Day 3) – linear model (after exclusion, BCa bootstrap applied)	−0.905 (0.396) *	[−1.734, −0.331]	0.027	0.226	23
**% change $SM_{RF-TRANSV}$ from day 1 to day 3, at lower third point**					
Weight [kg] at admission – linear model (after exclusion, BCa bootstrap applied)	0.986 (0.333) **	[0.291, 1.561]	0.006	0.241	24
SAS [points] (average over the first 3 days) – linear model (after exclusion, BCa bootstrap applied)	15.256 (7.151) *	[2.587, 26.874]	0.026	0.137	25
Each model was fitted using simple linear regression, with Δ% CSA_{RF}, Δ% $ECHO_{RF}$, or Δ% SM_{RF} as the dependent variable. Analyses were conducted for clinically relevant variables considered potentially impactful on skeletal muscle					

Table E15 – Univariate linear regressions between percentage changes in ultrasound-derived *rectus femoris* parameters and selected clinical variables.

tissue dysfunction during the acute and early phase of critical illness, including: *Age [years]; Height [cm]; Weight [kg]; BMI [kg/m²]; SOFA score [points] at admission; SAPS II [points] at admission; ICU length of stay [days]; SAS [points] (average over the first 3 days); total neuromuscular blocker dose [mg] (first 3 days); cumulative fluid balance [mL] (to day 5); caloric intake [kcal] (averaged over the first 3 days); protein intake [g] (average over the first 3 days); mean CRP [mg/l] (first 3 days); mean lactate [mmol/l] (first 3 days); mean glycaemia [mmol/l] (first 3 days); Δ% CSA_{RF} [cm²] (lower third, Day 1–Day 3); and Δ% $ECHO_{RF}$ [grey scale] (midpoint, Day 1–Day 3).*

Variables were retained for further diagnostic evaluation and presentation when the initial univariate model showed an association with the dependent variable at $p < 0.10$.

The 95% confidence intervals were computed as β ± 1.96 × SE; *p*-values correspond to two-tailed t-tests (α = 0.05). For CRP, lactate, and glycaemia, mean values for the first three ICU days were calculated when data from at least two days were available. "Highest daily" variables represent the mean of daily maxima across days 1–3. [§], $p < 0.10$; *, $p < 0.05$; **, $p < 0.01$; ***, $p < 0.001$

Abbreviations: BCa, bias-corrected and accelerated bootstrap; BMI, body mass index; CI, confidence interval; CRP, C-reactive protein; CSA_{RF}, cross-sectional area of the *rectus femoris*; $ECHO_{RF}$, echogenicity of the *rectus femoris*; ICU, intensive care unit; SAPS II, Simplified Acute Physiology Score II; SAS, Sedation–Agitation Scale; SE, standard error; SM_{RF}, shear modulus of the *rectus femoris*; SOFA, Sequential Organ Failure Assessment.

EXPLANATION:

- Δ% CSA_{RF} (Day 1–Day 5, lower third) - SOFA score at ICU admission

A simple linear regression was first fitted to assess the association between baseline SOFA score and the percentage change in rectus femoris cross-sectional area (CSA) between day 1 and day 5. The initial model suggested a weak positive slope (β = 6.949 ± 3.826, p = 0.086, R^2 = 0.155). Residual diagnostics indicated one highly influential observation, whose removal substantially altered the coefficient (β = 1.881 ± 2.700, *p* = 0.495, R^2 = 0.028), confirming that the apparent linear trend was driven by that single case. To examine potential non-linearity, a quadratic model was then fitted using standardised SOFA values. The inclusion of the squared term significantly improved model fit (R^2 = 0.395 after exclusion) and the quadratic component was significant ($β^2$ = 10.979 ± 3.527, *p* = 0.007). Residuals from this specification met normality (Shapiro–Wilk p = 0.127) and homoscedasticity assumptions, with no further influential points detected.

However, after re-estimation excluding the outlier and rechecking both terms, neither the linear (*p* = 0.782) nor the quadratic (*p* = 0.533) components remained significant, and model fit dropped (R^2 = 0.107). The final accepted specification was therefore the linear model after exclusion, which satisfied all standard regression assumptions (normality, homoscedasticity, independence).

- Δ% CSA_{RF} (Day 1–Day 5, lower third) - Mean protein intake (first 3 days)

A linear regression model was fitted to examine the association between mean protein intake during the first three ICU days and Δ% CSA_{RF} from day 1 to day 5. Diagnostic inspection identified one influential observation, whose exclusion improved model stability and assumption compliance. In the refined model (N = 19), the regression slope remained negative and approached statistical significance (B = −0.763 ± 0.405, *p* = 0.077, R^2 = 0.173).

Residual analysis confirmed satisfactory model assumptions. The Shapiro–Wilk test indicated no deviation from normality (W = 0.933, *p* = 0.199). The Glejser test revealed no evidence of heteroscedasticity (*p* = 0.446), and the

White test was also non-significant ($p = 0.409$), supporting homoscedasticity. No additional influential points were detected after exclusion.
A quadratic term was tested but did not significantly improve model fit ($p = 0.491$; $\Delta R^2 = 0.024$). The relationship was therefore considered adequately described by a linear model fulfilling all standard regression assumptions. Bias-corrected and accelerated (BCa) bootstrap estimation yielded comparable results ($B = -0.763 \pm 0.421$, $p = 0.096$, 95% CI [−1.661; 0.036]), confirming the robustness of the association under resampling.

- Δ% CSA_{RF} (Day 1–Day 5, lower third) - Highest daily glycaemia level (first 3 days)

A hierarchical linear regression approach was used to assess the association between Highest daily glycaemia level during the first three ICU days and the Δ% CSA_{RF} between day 1 and day 5. The initial model (N = 20) indicated a positive slope ($\beta = 4.565 \pm 2.558$, $p = 0.091$, $R^2 = 0.150$), but residual analysis revealed non-normality (Shapiro–Wilk $p = 0.009$) and one highly influential case. After removing this case, model assumptions were met (normality $p = 0.162$, homoscedasticity $p = 0.606$), and the slope reversed direction, becoming non-significant ($\beta = -0.936 \pm 2.015$, $p = 0.648$, $R^2 = 0.013$). A quadratic specification was tested ($\beta^2 = 7.85 \pm 3.18$, $p = 0.025$), but the apparent curvature did not improve overall model fit ($R^2 = 0.285$, $p = 0.068$) once assumptions were verified. The final accepted model was the linear specification after exclusion, which satisfied all regression assumptions and showed no evidence of influential bias or meaningful non-linearity.

- Δ% $ECHO_{RF}$ (Day 1–Day 5, midpoint) - Mean caloric intake (first 3 days)

A simple linear regression model was fitted to examine the association between mean caloric intake during the first three ICU days and Δ% $ECHO_{RF}$ at the midpoint site from day 1 to day 5. In the initial model (N = 20), the regression slope was positive and statistically significant ($\beta = 0.030 \pm 0.013$, $p = 0.032$, $R^2 = 0.230$). Diagnostic checks confirmed that residuals were approximately normally distributed and homoscedastic, with no influential cases detected. To assess potential non-linearity, a quadratic term was added, but this did not improve model fit (overall $p = 0.081$), and neither the linear nor the squared term remained significant in that specification. The final accepted model was therefore the simple linear regression, which met all standard assumptions and showed stable coefficients across diagnostic tests.

- Δ% $ECHO_{RF}$ (Day 1–Day 5, midpoint) - Mean lactate (first 3 days)

The association between mean lactate levels during the first three ICU days and Δ% $ECHO_{RF}$ was explored. In the initial model (N = 20), the linear coefficient was positive and significant ($\beta = 1.94$, $p = 0.017$); however, residual analysis revealed two influential observations, which markedly distorted the fit. After exclusion of these cases (N = 18), the linear effect disappeared ($p = 0.807$), and residuals satisfied normality and homoscedasticity assumptions. A quadratic model was then tested on the cleaned dataset and indicated a curvilinear trend (overall $p = 0.048$), consistent with a modest inverted-U relationship, although the squared term contributed minimally to explained variance and confidence intervals remained wide. Given the limited sample and the model's sensitivity to influential points, the final accepted specification was the linear model after exclusion, which provided a statistically valid fit without violating regression assumptions.

- Δ% $SM_{RF\text{-}LONG}$ (Day 1–Day 3, midpoint) - SOFA score at ICU admission

The association between day-1 SOFA and percentage change in Δ% $SM_{RF\text{-}LONG}$ (midpoint) from day 1 to day 3 was assessed. In the initial model (N = 21), the linear effect was positive and significant ($\beta = 18.212$, $p = 0.019$), but residual diagnostics revealed two influential observations and non-normal errors. After exclusion (N = 19), the linear

effect remained and became more precise (β = 18.178, $p = 0.002$; $R^2 = 0.436$) with no evidence of heteroscedasticity (Glejser p = 0.844; White $p = 0.820$), though normality was still marginal (Shapiro–Wilk p = 0.016). A quadratic specification on the cleaned data was globally significant ($F(2,16) = 6.637$, $p = 0.008$), yet the squared term was not (p = 0.485), indicating an essentially linear relationship.
To strengthen inference given small N and mild non-normality, a BCa bootstrap (5,000 samples) was applied to the final linear model: the bootstrap estimate matched the parametric β, with SE = 4.307, BCa 95% CI [10.010, 27.161], and p = 0.003, reinforcing the robust, linear association between SOFA (D1) and Δ% $SM_{RF-LONG}$ (D3–D1).

- Δ% $SM_{RF-LONG}$ (Day 1–Day 3, lower third) - SOFA score at ICU admission

The association between SOFA score at ICU admission and Δ% $SM_{RF-LONG}$ (D3–D1, lower third) was examined. In the initial linear model (N = 22), the coefficient was positive and significant (β = 13.893, p = 0.039); however, one influential observation was identified and excluded. In the final model (N = 21), the effect remained positive (β = 8.453, $p = 0.034$, $R^2 = 0.215$), with normally distributed residuals but evidence of mild heteroscedasticity (Glejser $p = 0.017$). A quadratic specification slightly improved fit (overall p = 0.037) but the squared term was not significant ($p = 0.141$), supporting a predominantly linear relationship. Given the small sample and presence of heteroscedasticity, a bias-corrected and accelerated (BCa) bootstrap procedure with 5,000 resamples was applied. The bootstrap estimate remained positive (β = 8.453, SE = 4.544), although the 95% BCa confidence interval included zero (−0.887 to 14.601, $p = 0.083$), indicating a modest and non-robust association.

- Δ% $SM_{RF-LONG}$ (Day 1–Day 3, lower third) – Cumulative fluid balance at Day 3

The association between cumulative fluid balance and Δ% $SM_{RF-LONG}$ (lower third) from day 1 to day 3 was examined. After removal of one influential case, the final linear model (N = 21) demonstrated a positive association (β = 3.316 % per litre, SE = 0.904, 95% CI [1.424, 5.209], $p = 0.002$; $R^2 = 0.414$). Although residual normality was not supported (Shapiro–Wilk p < .001), homoscedasticity was confirmed (Glejser $p = .424$; White $p = .185$). A bias-corrected and accelerated (BCa) bootstrap with 5,000 resamples yielded consistent results (β = 3.316, SE = 0.992, BCa 95% CI [1.124, 4.306], $p = 0.002$). Quadratic modelling (Z and Z^2) did not improve model fit. Each additional litre of cumulative positive fluid balance was associated with an approximately three-percentage-point increase in Δ% $SM_{RF-LONG}$ (lower third).

- Δ% $SM_{RF-TRANSV}$ (Day 1–Day 3, midpoint) – Mean caloric intake (first 3 days)

A linear regression analysis was conducted to evaluate whether mean caloric intake during the first three ICU days was associated with the Δ% $SM_{RF-TRANSV}$ (D3–D1, midpoint). In the initial model including all 24 participants, caloric intake showed a positive trend towards significance (β = 0.078, SE = 0.040, 95 % CI [−0.004, 0.161], $p = 0.062$; $R^2 = 0.150$). Inspection of diagnostic plots identified one influential observation. This case was removed in accordance with predefined influence criteria. The model was re-estimated on the remaining 23 participants, resulting in improved precision and model fit (β = 0.076, SE = 0.029, 95 % CI [0.016, 0.135], $p = 0.015$; $R^2 = 0.252$). Residuals did not meet the assumption of normality (Shapiro–Wilk $p = 0.003$), but tests for heteroscedasticity were non-significant (Glejser p = 0.583; White $p = 0.432$), and linearity was confirmed (quadratic term p = 0.802). No collinearity was detected (VIF = 1.00).
To account for non-normal residuals, a bias-corrected and accelerated (BCa) bootstrap with 5,000 resamples was performed. The bootstrap coefficient remained stable (β = 0.076, SE = 0.026, BCa 95 % CI [0.029,0.126], $p = 0.006$), supporting the robustness of the association.

Each 100 kcal/day increase in mean caloric intake was associated with an estimated 7.6 percentage-point change in Δ% $SM_{RF-TRANSV}$ (D3–D1, midpoint).

- Δ% $SM_{RF-TRANSV}$ (Day 1–Day 3, midpoint) – Mean CRP (first 3 days)

A simple linear regression was performed to examine the association between mean C-reactive protein (CRP) levels during the first three ICU days and Δ% $SM_{RF-TRANSV}$ (D3–D1, midpoint).
In the initial model including all 24 participants, CRP showed a significant positive association with Δ% Δ% $SM_{RF-TRANSV}$ (β = 0.197, SE = 0.080, 95% CI [0.031, 0.362], p = 0.022; R^2 = 0.216). Residual inspection revealed mild right-skewness, and the normality assumption was not fully supported (Shapiro–Wilk p = 0.015). Two observations were identified as influential and were excluded according to pre-specified diagnostic criteria. Re-estimation on the reduced sample (N = 22) yielded highly similar coefficients (β = 0.181, SE = 0.077, 95% CI [0.021–0.341], p = 0.029; R^2 = 0.217), indicating that the association was not driven by those points. The distribution of residuals improved substantially (Shapiro–Wilk p = 0.62), and linearity was confirmed (quadratic term p = 0.61).
A bias-corrected and accelerated (BCa) bootstrap was applied to the final model. Bootstrap estimates were nearly identical (β = 0.181, SE = 0.068, BCa 95% CI [0.050–0.335], p = 0.016), confirming the stability of the linear relationship.
Overall, each 1 mg/L increase in mean CRP was associated with an estimated 0.18 percentage-point change in Δ% $SM_{RF-TRANSV}$ (midpoint) between day 1 and day 3.

- Δ% $SM_{RF-TRANSV}$ (Day 1–Day 3, midpoint) – Δ% $ECHO_{RF}$ (D3–D1, midpoint)

A simple linear regression was performed to test whether the Δ% $ECHO_{RF}$ (D3–D1, midpoint) was associated with the percentage change in transverse shear modulus Δ% $SM_{RF-TRANSV}$ (D3–D1, midpoint). In the initial model (N = 24), the association was negative but not significant (β = −0.925, SE = 0.507, 95% CI [−1.976, 0.127], p = 0.082; R^2 = 0.131). Residuals showed non-normality (Shapiro–Wilk p ≈ 0.016), and one influential observation was detected.
A quadratic model including both the linear and squared terms for Δ% $ECHO_{RF}$ (D3–D1, midpoint) did not improve model fit (p = 0.856 for the quadratic term; overall F-test p = 0.224), supporting a linear specification. After exclusion of the single influential case (N = 23), the negative association became statistically significant (β = −0.905, SE = 0.365, 95% CI [−1.665, −0.146], p = 0.022; R^2 = 0.226). Standardised coefficients indicated a moderate effect (β standardised = −0.476). Residuals remained slightly non-normal (Shapiro–Wilk p = 0.016), justifying the use of robust inference. A bias-corrected and accelerated (BCa) bootstrap procedure confirmed the stability of the result (β = −0.905, SE = 0.396, BCa 95% CI [−1.734, −0.331], p = 0.027; R^2 = 0.226). Thus, a 1-percentage-point increase in Δ% $ECHO_{RF}$ (D3–D1, midpoint) was associated with an estimated 0.91-percentage-point decrease in Δ% $SM_{RF-TRANSV}$ (D3–D1, midpoint) between day 1 and day 3.

- Δ% $SM_{RF-TRANSV}$ (Day 1–Day 3, lower third) – Weight (at admission)

A simple linear regression was performed to examine the association between body weight (kg) and the Δ% $SM_{RF-TRANSV}$ (D3–D1, lower third). In the initial model (N = 25), the association was positive but not statistically significant (β = 0.750, SE = 0.436, 95% CI [−0.151, 1.652], p = 0.099; R^2 = 0.114). One influential observation was detected. After exclusion of this case (N = 24), the coefficient increased and reached statistical significance (β = 0.986, SE = 0.373, 95% CI [0.212–1.760], p = 0.015; R^2 = 0.241). Residual normality was supported in both the initial and final models (Shapiro–Wilk p = 0.354 and p = 0.311, respectively). The assumption of homoscedasticity was satisfied (Glejser p =

0.944), and collinearity was absent (VIF = 1.00). A quadratic specification including both the linear and squared terms for weight did not improve model fit ($p = 0.805$ with all cases; $p = 0.813$ without the influential case; $\Delta R^2 < 0.002$), confirming that the linear form was adequate.

To account for small-sample uncertainty, a bias-corrected and accelerated (BCa) bootstrap procedure was applied to the final model. The bootstrap estimate remained consistent ($\beta = 0.986$, SE = 0.333, BCa 95% CI [0.291, 1.561], $p = 0.006$; $R^2 = 0.241$). Each additional kilogram of body weight was associated with an estimated 0.99 percentage-point change in Δ% $SM_{RF\text{-}TRANSV}$ (D3–D1, lower third) between day 1 and day 3.

- Δ% $SM_{RF\text{-}TRANSV}$ (Day 1–Day 3, lower third) – Mean SAS (first 3 days)

A simple linear regression was conducted to assess whether the mean Sedation–Agitation Scale (SAS) score over the first three ICU days was associated with Δ% $SM_{RF\text{-}TRANSV}$ (D3–D1, lower third).

In the initial model including all 25 participants, the association was positive and approached statistical significance ($\beta = 15.256$, SE = 7.969, 95% CI [−1.228, 31.740], $p = 0.068$; $R^2 = 0.137$). Inspection of diagnostic indices revealed no influential observations, and assumptions of normality (Shapiro–Wilk $p > 0.05$) and homoscedasticity were satisfied.

A quadratic model including both the linear and squared terms for SAS did not improve the fit ($\Delta R^2 < 0.01$; all $p > 0.70$), confirming that the association was adequately represented by a linear relationship.

Given the marginal significance level and the small sample size, a bias-corrected and accelerated (BCa) bootstrap was conducted. The bootstrap results supported the stability of the effect ($\beta = 15.256$, SE = 7.151, BCa 95% CI [2.587, 26.874], $p = 0.026$; $R^2 = 0.137$), indicating a robust positive association.

Each one-point increase in mean SAS corresponded to an estimated 15-percentage-point change in Δ% $SM_{RF\text{-}TRANSV}$ (D3–D1, lower third) between day 1 and day 3.

Table E16 – Multivariate linear regressions between percentage changes in ultrasound-derived *rectus femoris* parameters and selected clinical variables.

Variable	β (SE)	95% CI	*p*-value	Standardised β	Tolerance	VIF	BCa 95% CI	*p*-value (BCa)
**% change CSA_{RF} from day 1 to day 5, at lower third point /** SOFA [points] and Protein [g]								
SOFA score [points], at admission	1.644 (2.538)	[−3.735, 7.024]	0.526	0.146	0.997	1.003	[−5.050, 5.936]	0.644
Protein intake [g] (average over the first 3 days)	−0.749 (0.413)	[−1.623, 0.126]	0.088	−0.408	0.997	1.003	[−1.673, 0.331]	0.112
Model: R^2 = 0.194; adjusted R^2 = 0.093; $F(2,16)$ = 1.922; *p* = 0.179; Durbin–Watson = 1.919; N = 19 (after exclusion of one influential case).<br>Diagnostics: residual normality (Shapiro–Wilk *p* = 0.106); homoscedasticity (Glejser *p* = 0.417; White *p* = 0.256); no multicollinearity (Tolerance ≥ 0.997; VIF ≤ 1.003; no remaining influential cases.								
**% change CSA_{RF} from day 1 to day 5, at lower third point /** SOFA [points] and Highest daily glycaemia level [mmol/l]								
SOFA score [points], at admission	−2.130 (3.208)	[−9.010, 4.751]	.518	−0.176	0.979	1.021	[−10.956, 5.961]	.437
Highest daily glycaemia level [mmol/l] (average over the first 3 days)	0.495 (1.796)	[−3.357, 4.347]	.787	0.073	0.979	1.021	[−5.964, 3.714]	.836
Model: R^2 = 0.040; adjusted R^2 = −0.097; $F(2,14)$ = 0.291; *p* = 0.752; Durbin–Watson = 1.069; N = 17 (after exclusion of three influential cases).<br>Diagnostics: residual normality (Shapiro–Wilk *p* = 0.740); homoscedasticity (Glejser *p* = 0.865; White *p* = 0.956); no multicollinearity (Tolerance ≥ 0.979; VIF ≤ 1.021); no remaining influential cases.								
**% change CSA_{RF} from day 1 to day 5, at lower third point /** Protein [g] and Highest daily glycaemia level [mmol/l]								
Protein intake [g] (average over the first 3 days)	−0.768 (0.436)	[−1.693, 0.157]	0.098	−0.418	0.915	1.093	[−1.862, 0.075]	0.105
Highest daily glycaemia level [mmol/l] (average over the first 3 days)	0.081 (1.987)	[−4.130, 4.293]	0.968	0.010	0.915	1.093	[−6.691, 3.116]	0.981
Model: R^2 = 0.173; adjusted R^2 = 0.069; $F(2,16)$ = 1.670; *p* = 0.219; Durbin–Watson = 1.814; N = 19 (after exclusion of one influential case).<br>Diagnostics: residual normality (Shapiro–Wilk *p* = 0.203); homoscedasticity (Glejser *p* = 0.430; White *p* = 0.399); no multicollinearity (Tolerance ≥ 0.915; VIF ≤ 1.093); no remaining influential cases.								
**% change $ECHO_{RF}$ from day 1 to day 5, at midpoint /** Caloric intake [kcal] and Mean Lactate [mmol/l]								
Caloric intake [kcal] (average first 3 days)	0.003 (0.013)	[−0.023, 0.030]	0.788	0.072	0.950	1.053	[−0.021, 0.028]	0.788

**Table E16** – Multivariate linear regressions between percentage changes in ultrasound-derived *rectus femoris* parameters and selected clinical variables.								
**Variable**	**β (SE)**	**95% CI**	***p*-value**	**Standardised β**	**Tolerance**	**VIF**	**BCa 95% CI**	***p*-value (BCa)**
Mean Lactate [mmol/l] (average first 3 days)	0.403 (2.321)	[−4.543, 5.350]	0.864	0.046	0.950	1.053	[−4.595, 6.706]	0.890
Model: $R^2 = 0.009$; adjusted $R^2 = -0.123$; $F(2,15) = 0.066$; $p = 0.936$; Durbin–Watson = 2.640; N = 18 (after exclusion of two influential cases). Diagnostics: residual normality (Shapiro–Wilk $p = 0.156$); homoscedasticity (Glejser $p = 0.565$; White $p = 0.844$); no multicollinearity (Tolerance ≥ 0.950; VIF ≤ 1.053); no remaining influential cases.								
**% change $SM_{RF\text{-}LONG}$ from day 1 to day 3, at lower third point** / SOFA [points] and Cumulative fluid balance [Litres]								
SOFA score [points], at admission	3.303 (3.799)	[−4.711, 11.318]	0.397	0.192	0.999	1.001	[−5.496, 10.557]	0.404
Cumulative fluid balance [Litres] (cumulative to day 3)	2.164 (1.338)	[−0.659, 4.987]	0.124	0.358	0.999	1.001	[−0.140, 5.041]	0.065
Model: $R^2 = 0.169$; adjusted $R^2 = 0.071$; $F(2,17) = 1.725$; $p = 0.208$; Durbin–Watson = 2.610; N = 20 (after exclusion of two influential cases). Diagnostics: residual normality (Shapiro–Wilk $p = 0.411$); homoscedasticity (Glejser $p = 0.521$; White $p = 0.559$); no multicollinearity (Tolerance ≥ 0.999; VIF ≤ 1.001); no remaining influential cases.								
**% change $SM_{RF\text{-}TRANSV}$ from day 1 to day 3, at midpoint** / Caloric intake [kcal] and CRP [mg/l]								
**Variable**	**β (SE)**	**95% CI**	***p*-value**	**Standardised β**	**Tolerance**	**VIF**	**BCa 95% CI**	***p*-value (BCa)**
Caloric intake [kcal] (average first 3 days)	0.074 (0.024)	[0.024, 0.123]	0.006	0.487	0.999	1.001	[0.028, 0.126]	0.003
CRP [mg/l] (average first 3 days)	0.158 (0.050)	[0.052, 0.263]	0.005	0.496	0.999	1.001	[0.062, 0.289]	0.003
Model: $R^2 = 0.497$; adjusted $R^2 = 0.447$; $F(2,20) = 9.886$; $p = 0.001$; Durbin–Watson = 1.461; N = 23 (after exclusion of one influential case). Diagnostics: residual normality (Shapiro–Wilk $p = 0.308$); homoscedasticity (Glejser $p = 0.481$; White $p = 0.451$); no multicollinearity (Tolerance ≥ 0.999; VIF ≤ 1.001); no remaining influential cases.								
**% change $SM_{RF\text{-}TRANSV}$ from day 1 to day 3, at midpoint** / Caloric intake [kcal] and % change $ECHO_{RF}$ (D1-D3, midpoint)								
Caloric intake [kcal] (average first 3 days)	0.081 (0.024)	[0.032, 0.131]	0.003	0.539	0.995	1.005	[0.047, 0.120]	< 0.001
% change $ECHO_{RF}$ (Day 1–Day 3, midpoint)	−0.979 (0.297)	[−1.598, −0.359]	0.004	−0.514	0.995	1.005	[−1.688, −0.493]	0.011

**Table E16** – Multivariate linear regressions between percentage changes in ultrasound-derived *rectus femoris* parameters and selected clinical variables.								
**Variable**	**β (SE)**	**95% CI**	***p*-value**	**Standardised β**	**Tolerance**	**VIF**	**BCa 95% CI**	***p*-value (BCa)**
Model: $R^2 = 0.515$; adjusted $R^2 = 0.467$; $F(2,20) = 10.619$; $p < 0.001$; Durbin–Watson = 1.690; N = 23 (after exclusion of one influential case). Diagnostics: residual normality (Shapiro–Wilk p = 0.441); homoscedasticity (Glejser $p = 0.439$; White $p = 0.683$); no multicollinearity (Tolerance ≥ 0.995; VIF ≤ 1.005); no remaining influential cases.								
**% change $SM_{RF\text{-}TRANSV}$ from day 1 to day 3, at midpoint** / CRP [mg/l] and % change $ECHO_{RF}$ (D1-D3, midpoint)								
CRP [mg/l] (average first 3 days)	0.158 (0.072)	[0.006, 0.309]	0.042	0.407	0.975	1.026	[0.054, 0.299]	0.012
% change $ECHO_{RF}$ (Day 1–Day 3, midpoint)	−0.716 (0.355)	[−1.459, 0.026]	0.058	−0.377	0.975	1.026	[−1.448, 0.061]	0.042
Model: $R^2 = 0.356$; adjusted $R^2 = 0.288$; $F(2,19) = 5.243$; $p = 0.015$; Durbin–Watson = 1.543; N = 22 (after exclusion of two influential cases). Diagnostics: residual normality (Shapiro–Wilk $p = 0.933$); homoscedasticity (Glejser $p = 0.180$; White $p = 0.312$); no multicollinearity (Tolerance ≥ 0.975; VIF ≤ 1.026); no remaining influential cases.								
**% change $SM_{RF\text{-}TRANSV}$ from day 1 to day 3, at lower third point** / Weight [kg] and SAS [points]								
Weight [kg] at admission	0.664 (0.367)	[-0.100, 1.429]	0.085	0.334	0.970	1.031	[0.016, 1.112]	0.046
SAS [points] (average over the first 3 days)	14.612 (6.306)	[1.457, 27.766]	0.031	0.427	0.970	1.031	[-3.657, 26.283]	0.016
Model: $R^2 = 0.342$; adjusted $R^2 = 0.277$; $F(2,20) = 5.207$; $p = 0.015$; Durbin–Watson = 2.540; N = 23 (after exclusion of two influential cases). Diagnostics: residual normality (Shapiro–Wilk $p = 0.835$); homoscedasticity (Glejser $p = 0.562$; White $p = 0.922$); no multicollinearity (Tolerance ≥ 0.970; VIF ≤ 1.031); no remaining influential cases.								

Each model was fitted using multiple linear regression, with Δ% CSA_{RF}, Δ% $ECHO_{RF}$, or Δ% SM_{RF} as the dependent variable. Independent variables were selected from those showing an association at $p < 0.10$ in the univariate analyses and were entered in pairs to explore potential additive or interactive effects. This strategy aimed to limit model overfitting given the sample size while ensuring clinical interpretability. For each model, regression diagnostics were performed, including assessment of normality (Shapiro–Wilk test), homoscedasticity (Glejser and White tests), and multicollinearity (tolerance and VIF). BCa bootstrap confidence intervals (5,000 resamples) were calculated when appropriate.

The 95% confidence intervals were computed as β ± 1.96 × SE; p-values correspond to two-tailed t-tests (α = 0.05). For CRP, lactate, and glycaemia, mean values for the first three ICU days were calculated when data from at least two days were available. “Highest daily” variables represent the mean of daily maxima across days 1–3. §, $p < 0.10$; *, $p < 0.05$; **, $p < 0.01$; ***, $p < 0.001$.

**Table E16** – Multivariate linear regressions between percentage changes in ultrasound-derived *rectus femoris* parameters and selected clinical variables.								
**Variable**	**β (SE)**	**95% CI**	***p*-value**	**Standardised β**	**Tolerance**	**VIF**	**BCa 95% CI**	***p*-value (BCa)**

Abbreviations: BCa, bias-corrected and accelerated; CRP, C-reactive protein; CSA_{RF}, cross-sectional area of the rectus femoris; $ECHO_{RF}$, echogenicity of the rectus femoris; ICU, intensive care unit; SAS, Sedation–Agitation Scale; SE, standard error; SM_{RF}, shear modulus of the rectus femoris; SOFA, Sequential Organ Failure Assessment; VIF, variance inflation factor.

BIBLIOGRAPHY

1. Hasselman, C.T., Best, T.M., Hughes, C.t., Martinez, S., and Garrett, W.E., Jr., *An explanation for various rectus femoris strain injuries using previously undescribed muscle architecture.* Am J Sports Med, 1995. **23**(4): p. 493-9.
2. Gruther, W., Benesch, T., Zorn, C., Paternostro-Sluga, T., Quittan, M., Fialka-Moser, V., Spiss, C., Kainberger, F., and Crevenna, R., *Muscle wasting in intensive care patients: Ultrasound observation of the M. quadriceps femoris muscle layer.* Journal of Rehabilitation Medicine, 2008. **40**(3): p. 185-189.
3. Pillen, S., *Skeletal muscle ultrasound.* European Journal of Translational Myology, 2010. **20**(4): p. 145-156.
4. Puthucheary, Z.A., Phadke, R., Rawal, J., McPhail, M.J., Sidhu, P.S., Rowlerson, A., Moxham, J., Harridge, S., Hart, N., and Montgomery, H.E., *Qualitative Ultrasound in Acute Critical Illness Muscle Wasting.* Crit Care Med, 2015. **43**(8): p. 1603-11.
5. Creze, M., Nordez, A., Soubeyrand, M., Rocher, L., Maitre, X., and Bellin, M.F., *Shear wave sonoelastography of skeletal muscle: basic principles, biomechanical concepts, clinical applications, and future perspectives.* Skeletal Radiol, 2018. **47**(4): p. 457-471.
6. Gennisson, J.L., Deffieux, T., Fink, M., and Tanter, M., *Ultrasound elastography: Principles and techniques.* Diagnostic and Interventional Imaging, 2013. **94**(5): p. 487-495.
7. De Jonghe, B., Sharshar, T., Lefaucheur, J.P., Authier, F.J., Durand-Zaleski, I., Boussarsar, M., Cerf, C., Renaud, E., Mesrati, F., Carlet, J., Raphael, J.C., Outin, H., Bastuji-Garin, S., and Groupe de Reflexion et d'Etude des Neuromyopathies en, R., *Paresis acquired in the intensive care unit: a prospective multicenter study.* JAMA, 2002. **288**(22): p. 2859-67.
8. Gosselink, R., Clerckx, B., Robbeets, C., Vanhullebusch, T., Vanpee, G., and Segers, J., *Physiotherapy in the intensive care unit.* Neth J Crit Care, 2011. **15**(2): p. 66-75.
9. Kress, J.P. and Hall, J.B., *ICU-acquired weakness and recovery from critical illness.* N Engl J Med, 2014. **370**(17): p. 1626-35.
10. Hermans, G. and Van den Berghe, G., *Clinical review: intensive care unit acquired weakness.* Crit Care, 2015. **19**: p. 274.
11. Fan, E., Cheek, F., Chlan, L., Gosselink, R., Hart, N., Herridge, M.S., Hopkins, R.O., Hough, C.L., Kress, J.P., Latronico, N., Moss, M., Needham, D.M., Rich, M.M., Stevens, R.D., Wilson, K.C., Winkelman, C., Zochodne, D.W., Ali, N.A., ATS Committee on ICU-acquired Weakness in Adults, and American Thoracic Society, *An official American Thoracic Society Clinical Practice guideline: the diagnosis of intensive care unit-acquired weakness in adults.* Am J Respir Crit Care Med, 2014. **190**(12): p. 1437-46.
12. Ciesla, N., Dinglas, V., Fan, E., Kho, M., Kuramoto, J., and Needham, D., *Manual muscle testing: a method of measuring extremity muscle strength applied to critically ill patients.* J Vis Exp, 2011(50): p. e2632.
13. De Jonghe, B., Bastuji-Garin, S., Sharshar, T., Outin, H., and Brochard, L., *Does ICU-acquired paresis lengthen weaning from mechanical ventilation?* Intensive Care Med, 2004. **30**(6): p. 1117-21.

14. Jonghe, B.D., Bastuji-Garin, S., Durand, M.-C., Malissin, I., Rodrigues, P., Cerf, C., Outin, H., Sharshar, T., and Réanimation, G.d.R.e.d.E.d.N.E., *Respiratory weakness is associated with limb weakness and delayed weaning in critical illness*.* Critical Care Medicine, 2007. **35**(9): p. 2007-2015.
15. Ali, N.A., O'Brien, J.M., Hoffmann, S.P., Phillips, G., Garland, A., Finley, J.C.W., Almoosa, K., Hejal, R., Wolf, K.M., Lemeshow, S., Connors, J.F., and Marsh, C.B., *Acquired Weakness, Handgrip Strength, and Mortality in Critically Ill Patients.* American Journal of Respiratory and Critical Care Medicine, 2008. **178**(3): p. 261-268.
16. Hermans, G., Clerckx, B., Vanhullebusch, T., Segers, J., Vanpee, G., Robbeets, C., Casaer, M.P., Wouters, P., Gosselink, R., and Berghe, G.V.D., *Interobserver agreement of medical research council sum-score and handgrip strength in the intensive care unit.* Muscle & Nerve, 2012. **45**(1): p. 18-25.
17. Connolly, B.A., Jones, G.D., Curtis, A.A., Murphy, P.B., Douiri, A., Hopkinson, N.S., Polkey, M.I., Moxham, J., and Hart, N., *Clinical predictive value of manual muscle strength testing during critical illness: an observational cohort study.* Critical Care, 2013. **17**: p. R229.
18. Harris, P.A., Taylor, R., Thielke, R., Payne, J., Gonzalez, N., and Conde, J.G., *Research electronic data capture (REDCap)--a metadata-driven methodology and workflow process for providing translational research informatics support.* J Biomed Inform, 2009. **42**(2): p. 377-81.
19. Harris, P.A., Taylor, R., Minor, B.L., Elliott, V., Fernandez, M., O'Neal, L., McLeod, L., Delacqua, G., Delacqua, F., Kirby, J., Duda, S.N., and Consortium, R.E., *The REDCap consortium: Building an international community of software platform partners.* J Biomed Inform, 2019. **95**: p. 103208.
20. Xu, G.X., Chen, P.Y., Jiang, X., and Huang, C.C., *Visualization of Human Skeletal Muscle Mechanical Anisotropy by Using Dual-Direction Shear Wave Imaging.* IEEE Trans Biomed Eng, 2022. **69**(9): p. 2745-2754.
21. Cortez, C.D., Hermitte, L., Ramain, A., Mesmann, C., Lefort, T., and Pialat, J.B., *Ultrasound shear wave velocity in skeletal muscle: A reproducibility study.* Diagn Interv Imaging, 2016. **97**(1): p. 71-9.
